# AMEND 2.0: module identification and multi-omic data integration with multiplex-heterogeneous graphs

**DOI:** 10.1186/s12859-025-06063-x

**Published:** 2025-02-05

**Authors:** Samuel S. Boyd, Chad Slawson, Jeffrey A. Thompson

**Affiliations:** 1 Department of Biostatistics and Data Science, University of Kansas Medical Center, Kansas City, KS 66160 USA; 2 Department of Biochemistry, University of Kansas Medical Center, Kansas City, KS 66160 USA; 3 University of Kansas Cancer Center, Kansas City, KS 66160 USA; 4 University of Kansas Alzheimer’s Disease Research Center, Fairway, KS 66205 USA

**Keywords:** Biological networks, Active module identification, Multi-omic data integration

## Abstract

**Background:**

Multi-omic studies provide comprehensive insight into biological systems by evaluating cellular changes between normal and pathological conditions at multiple levels of measurement. Biological networks, which represent interactions or associations between biomolecules, have been highly effective in facilitating omic analysis. However, current network-based methods lack generalizability to accommodate multiple data types across a range of diverse experiments.

**Results:**

We present AMEND 2.0, an updated active module identification method which can analyze multiplex and/or heterogeneous networks integrated with multi-omic data in a highly generalizable framework, in contrast to existing methods, which are mostly appropriate for at most two specific omic types. It is powered by Random Walk with Restart for multiplex-heterogeneous networks, with additional capabilities including degree bias adjustment and biased random walk for multi-objective module identification. AMEND was applied to two real-world multi-omic datasets: renal cell carcinoma data from The cancer genome atlas and an O-GlcNAc Transferase knockout study. Additional analyses investigate the performance of various subroutines of AMEND on tasks of node ranking and degree bias adjustment.

**Conclusions:**

While the analysis of multi-omic datasets in a network context is poised to provide deeper understanding of health and disease, new methods are required to fully take advantage of this increasingly complex data. The current study combines several network analysis techniques into a single versatile method for analyzing biological networks with multi-omic data that can be applied in many diverse scenarios. Software is freely available in the R programming language at https://github.com/samboyd0/AMEND.

**Supplementary Information:**

The online version contains supplementary material available at 10.1186/s12859-025-06063-x.

## Introduction

High-throughput technologies provide granular information on molecular activity, offering insight into the mechanisms that drive biological processes and diseases. Omics data derived from these technologies have led to increased understanding of biological systems [1–3]. Given the complex regulatory mechanisms underlying cellular activity, which span from epigenomic to post-translational modifications (PTM), it is advantageous to obtain measurements on multiple molecular types to provide a more holistic view of this multifaceted landscape. Multi-omic data are increasingly being employed in biological research, which simultaneously addresses limitations inherent to single omic technologies and presents novel problems of data integration. Varying coverages, different data distributions, missingness, and noise present obstacles to the joint analysis of multi-omic data [4].

Molecular interaction networks (or graphs) have proved highly effective in their ability to decipher omics data [5, 6]. These networks contain nodes, which represent biological entities (e.g., proteins), and edges, which represent associations between entities (e.g., participation in a shared signaling pathway). Molecular measurements can be mapped to nodes and edges of the network to which graph-theoretic tools can be applied for diverse tasks including feature ranking [7], patient stratification [8], active module identification [9, 10], and data integration [11]. Many network-based methods rely on network diffusion, a mathematical framework for determining node importance in which prior knowledge of node importance is quantified and diffused across the network such that large diffusion scores reflect node proximity in the network to nodes who themselves are proximal to nodes with large prior importance [12]. Network diffusion approaches have been widely applied to the problem of multi-omic data integration, with most methods implementing the integration step prior to or after diffusion [13]. However, this precludes the direct influence of nodes of one data type on the diffusion scores of nodes of another data type. Additional pre- or post-processing steps, required to ensure proper integration before or after diffusion and often tailored to specific combinations of omics data, also limit these methods’ generalizability. Conversely, integration during diffusion only requires that the data be transformed to node-wise, stochastic vectors (a requirement inherent to all diffusion methods).

Network-based multi-omic data integration necessitates the creation of more complex graphs to which the data can be mapped. We employ the terms *multiplex* and *heterogeneous* to describe these complex graphs. A multiplex graph comprises several *layers*, which are subgraphs sharing a common node type but having different edge types or different data types mapped to nodes. This diverges slightly from common usage of *multiplex*, which traditionally refers to a graph with layers representing different edge types only. For example, a multiplex protein–protein interaction (PPI) network could contain two layers: one containing physical interactions between proteins, and the other containing functional associations between proteins, with edges linking common proteins to connect the layers. A heterogeneous graph consists of several *components*, which are subgraphs representing one of several node types. A heterogeneous graph can consist of both mono- and multi-plex components. For example, a heterogeneous graph could contain a PPI component and a metabolite-metabolite interaction (MMI) component, with protein-metabolite edges connecting them.

**Fig. 1:**
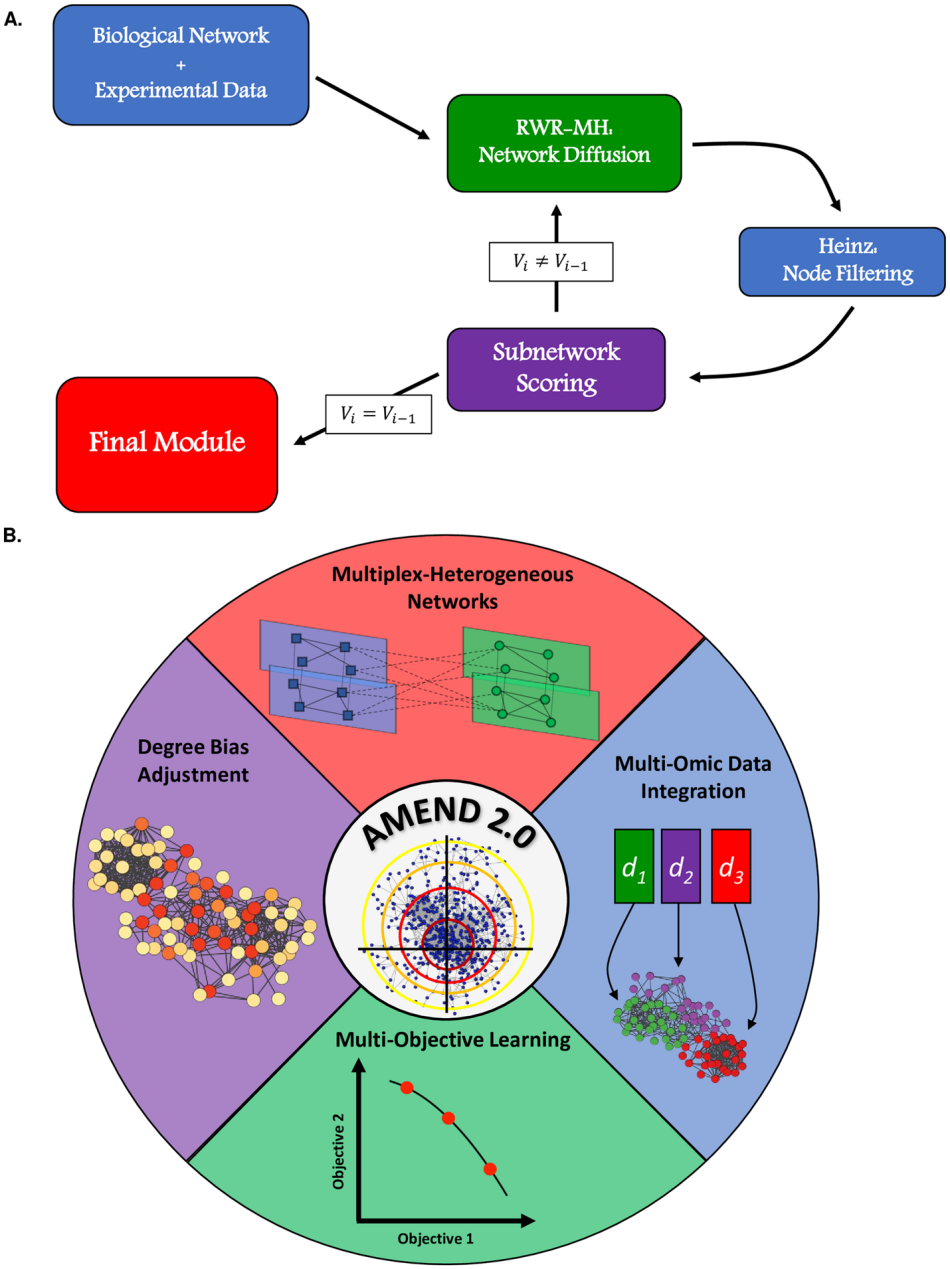
AMEND Capabilities and Workflow: **A** The basic workflow of the AMEND algorithm. It finds successively smaller subnetworks that are optimal in terms of experimental and connectivity information, stopping when the subnetwork reaches a user-defined size. **B** The newest version of the AMEND algorithm offers several new features, including methods for multiplex-heterogeneous graphs, biased random walk, and degree bias mitigation

This paper re-introduces the AMEND algorithm (Active Module identification with Experimental data and Network Diffusion), an iterative active module identification method that obtains node weights through network diffusion, filters out low-weight nodes to get a subnetwork, scores the subnetwork based on experimental and topological information, and then uses this subnetwork for the next iteration until an optimal subnetwork is found (Fig. 1A) [9]. AMEND is now equipped with Random Walk with Restart for Multiplex-Heterogeneous Graphs (RWR-MH), a versatile network diffusion method that allows for seamless multi-omic data integration on multiplex-heterogeneous graphs with fine control over integration dynamics. Further improvements of AMEND include multi-objective learning through a biased random walk process and degree bias mitigation to attenuate the influence of ‘hub’ nodes in the network (Fig. 1B). These new capabilities are tested on tasks of node ranking, degree bias adjustment, and active module identification using pathway data, gene expression data, and multi-omic datasets from The Cancer Genome Atlas renal cell carcinoma project (TCGA-KIRC) [14] and an O-GlcNAc Transferase (OGT) knockout study. Together, these features make AMEND a highly generalizable tool that can accommodate a wide range of molecular interaction networks and multi-omic datasets.

## Materials and methods

### Random walk with restart for multiplex/heterogeneous networks

Random Walks (RW) are mathematical models of stochastic processes on graphs. They allow a probabilistic characterization of the location of a ‘random walker’, a hypothetical agent that traverses the graph according to transition probabilities in a discrete-time process until convergence. Higher probabilities of the agent being at a node reflect higher importance. For RWs on graphs, there exist well-defined probabilities of transitioning between any node pair. These are usually defined by graph topology and do not depend on the history of the random walker’s trajectory in the graph, making RWs instances of Markov chains. These transition probabilities for a graph with $N$ nodes are represented by the transition matrix ${M}_{N\times N}=({m}_{ij})$ whose $i{j}^{th}$ element gives the probability of the random walker transitioning from node $j$ to node$i$, with columns summing to one. The transition matrix is usually constructed by column-normalizing the adjacency matrix $A=({a}_{ij})$ of the graph. For a diagonal matrix$D$, containing column sums of$A$, transition matrix $M$ can be defined as $M=A{D}^{-1}$. For prior probabilities of the random walker starting on each node, given by the stochastic vector${{\varvec{p}}}_{0}$, also called the seed vector, the evolution of a RW can formally be defined by iterative matrix multiplication, given by

1 $${\varvec{p}}_{i} = M{\varvec{p}}_{i - 1}$$

This formula is used to update ${{\varvec{p}}}_{i}$ until convergence. The seed vector represents a priori knowledge of node importance and often requires normalization. This discrete process converges to a stationary probability ${{\varvec{p}}}_{SD}^{T}=[{s}_{1},\dots ,{s}_{N}]$, where ${s}_{j}=\frac{{\sum }_{i}{a}_{ij}}{{\sum }_{i}{\sum }_{k}{a}_{ik}}$, regardless of the starting vector ${{\varvec{p}}}_{0}$ [15]. Therefore, stationary distribution probabilities are functions of node degree, and their independence from the seed vector in classic RW necessitates alterations to the updating formula if we want seed vectors to be relevant.

Random Walk with Restart (RWR) is a modification of RW [16]. It introduces the restart probability, which is the probability of the agent ‘restarting’ the random walk on its starting node. It governs the extent of network smoothing of the seed values. This is mathematically represented as

2 $${\varvec{p}}_{i} = \left( {1 - r} \right)M{\varvec{p}}_{i - 1} + r{\varvec{p}}_{0}$$

with $r$ as the restart probability parameter.

While this formulation of RWR is appropriate for simple graphs, it is not sufficient for more complex graphs comprising multiple node, edge, or seed value types. We employ the terms *multiplex* and *heterogeneous* to describe these complex graphs. A multiplex graph comprises several *layers*, which are graphs sharing a common node type but having different edge types or different seed value types. A heterogeneous graph consists of several *components*, which are graphs representing one of several node types. A heterogeneous graph can consist of both mono- and multi-plex components.

To account for this increased complexity, new protocols for constructing the transition matrix and seed vector are warranted. Without modification, Eq. 2 would cause seed vectors of different sizes and potentially disparate data types to be normalized together, in addition to the disruption of local diffusion dynamics by normalizing the graph adjacency matrix as a whole rather than each layer and component independently. RWR was generalized to heterogeneous graphs of two components by Li and Patra (2010), and further generalized to multiplex-heterogeneous graphs of two components—one monoplex and one multiplex—by Valdeolivas et al. (2019) This was improved upon by Baptista et al. (2022) with MultiXrank, which performs RWR on multiplex-heterogeneous graphs with arbitrary numbers of components and layers. These methods introduce two new classes of parameters to RWR; seed weight parameters govern the weight given to the seed vectors of individual layers and components, while cross-talk parameters, or jump/switch probabilities, control the extent of cross-talk between different layers and components by specifying the probability of the random walker ‘jumping’ from its current layer/component to another by taking a bipartite edge. Despite its improvements, MultiXrank does not calculate transition rates by normalizing layers of multiplex components independently, which disrupts local diffusion dynamics. Furthermore, their parameterization leads to inconsistent behavior across the parameter space for cross-talk parameters (see *Results*).

**Fig. 2:**
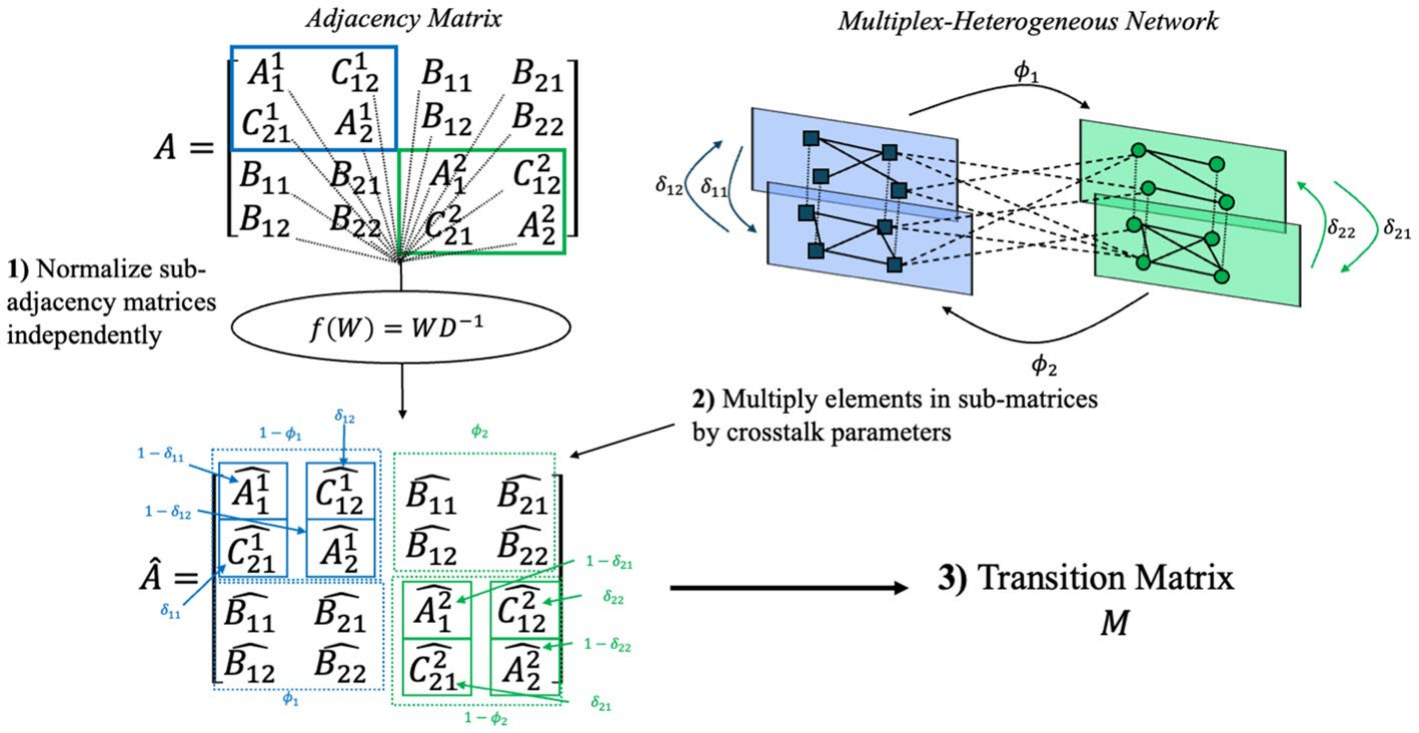
Transition matrix construction: diagram outlining the major steps in transition matrix construction for RWR-MH. See Additional File 1 for more details

Here we briefly present a re-parameterization of RWR for multiplex/heterogeneous graphs that includes further generalizations compared to MultiXrank, which we call RWR-MH. The essential difference is in how the transition matrix is constructed from the adjacency matrix. First, the intra-layer, inter-layer, and inter-component adjacency matrices are column-normalized independently (Fig. 2). Then, crosstalk parameters are applied to the transformed matrix such that all columns sum to one. Refer to Additional File 1 for more detailed information on the transition matrix construction process.

In addition to the new protocol for transition matrix construction, RWR-MH also involves further generalizations for the seed vector ${{\varvec{p}}}_{0}$, which is defined as

3 $${\varvec{p}}_{0}^{T} = \left[ {\begin{array}{*{20}c} {\eta_{1} \overline{\user2{u}}_{1}^{T} } & {\eta_{2} \overline{\user2{u}}_{2}^{T} } & \cdots & {\eta_{N} \overline{\user2{u}}_{N}^{T} } \\ \end{array} } \right]$$

where  ${\overline{{\varvec{u}}} }_{\kappa }^{T}$ represents the seed vector for component $\kappa$ with seed weight parameter ${\eta }_{\kappa }$, under the constraint ${\sum }_{i=1}^{N}{\eta }_{i}=1, {\eta }_{i}\ge 0 \forall i$. Component-wise seed vectors are further defined by

4 $$\overline{\user2{u}}_{\kappa }^{T} = \left[ {\begin{array}{*{20}c} {\tau_{\kappa 1} {\varvec{u}}_{\kappa 1}^{T} } & {\tau_{\kappa 2} {\varvec{u}}_{\kappa 2}^{T} } & \cdots & {\tau_{{\kappa L_{\kappa } }} {\varvec{u}}_{{\kappa L_{\kappa } }}^{T} } \\ \end{array} } \right]$$

where ${{\varvec{\mu}}}_{\kappa l}^{T}$ is the seed vector of layer $l$, component $\kappa$ with seed weight parameter ${\tau }_{\kappa l}$. These are under the constraints ${\sum }_{i=1}^{{L}_{\kappa }}{\tau }_{\kappa i}=1, {\tau }_{\kappa i}\ge 0 \forall i$, and ${\sum }_{i=1}^{{n}_{\kappa l}}{\left({{\varvec{u}}}_{\kappa l}\right)}_{i}=1,{\left({{\varvec{u}}}_{\kappa l}\right)}_{i}\ge 0 \forall i$. This allows different data types to be normalized independently and mapped to each layer and also enables control over their relative importance, thereby facilitating multi-omic integration. Following these steps for transition matrix and seed vector construction enables the same updating formula in Eq. 2 to be used for multiplex-heterogeneous graphs of an arbitrary number of components and layers. RWR-MH is embedded in the AMEND algorithm.

### Biased random walk

A biased random walk (BRW) is any random walk in which there are non-uniform transition probabilities from a node to its neighbors. For our purposes, the definition of a biased random walk is restricted to be a random walk in which node-wise, non-negative values are applied to the adjacency matrix in such a way that nodes with larger values have increased incoming transition probabilities. Given an adjacency matrix ${A}_{N\times N}=({a}_{ij})$ and a vector of non-negative values ${{\varvec{\mu}}}^{T}=[{\mu }_{1},\dots ,{\mu }_{N}]$, $A$ is right-multiplied by a diagonal matrix $B$ with elements from ${\varvec{\mu}}$ to yield

5 $$A^\prime = BA$$

This matrix ${A}{\prime}$ is then column-normalized to create transition matrix $M$ whose $i{j}^{th}$ element gives the probability of transitioning from node $j$ to node $i$ and is denoted as

6 $$m_{ij} = \frac{{\mu_{i} a_{ij} }}{{\mathop \sum \nolimits_{l} \mu_{l} a_{lj} }}$$

Therefore, the value ${\mu }_{i}$ increases the transition rate from node $j$ to node $i$ relative to the $\mu$ values of the neighbors of node $j$. In other words, the extent to which ${\varvec{\mu}}$ increases transition rates to target nodes depends on the local neighborhood of the source node. The vector ${\varvec{\mu}}$ can also be used to incorporate negative evidence to decrease transition rates to undesirable nodes. BRW can easily be extended to Biased Random Walk with Restart (B-RWR), which follows the same steps as above for transition matrix calculation. By incorporating both the seed vector ${{\varvec{p}}}_{0}$ and the BRW attribute vector ${\varvec{\mu}}$, B-RWR becomes a multi-objective learning algorithm.

B-RWR is embedded in the AMEND algorithm, alongside RWR-MH, in two distinct ways. First, users can explicitly provide a non-negative, numeric vector representing ${\varvec{\mu}}$. Second, users can provide nodes of interest, represented by node set $V$, from which ${\varvec{\mu}}$ is set to be a decreasing function of distance in the graph from nodes in $V$, thereby favoring transitions to nodes that are closer to these nodes of interest. In this scenario, the ${i}^{th}$ element of ${\varvec{\mu}}$ is given by

7 $$\mu_{i} = e^{{ - k \times d_{i} }}$$

where ${d}_{i}$ is the mean distance from node $i$ to nodes in $V$, and $k$ is a scaling factor.

### Degree bias adjustment methods

In general, degree is highly relevant for determining node importance, and methods such as RWR learn from node degree by design. However, in the context of PPI networks, which suffer from technical and study biases, degree is a corrupt metric, and this calls for the development of methods that attenuate degree influence. Although various permutation-based approaches have been introduced [7, 17], these are too computationally intensive for use in the iterative AMEND algorithm. This study compares three degree bias adjustment methods: Stationary Distribution Scaling (SDS), Bistochastic Scaling (BS), and Inflation-Normalization (IN). All three rely on the logic that the stationary distribution of a transition matrix is a good proxy for the amount of degree influence on the diffusion scores of each node. This is justified since the stationary distribution probability is a function of node degree.

SDS, previously introduced as the Eigenvector Centrality method by Erten et al. (2011), involves scaling diffusion scores by stationary distribution probabilities. The modified diffusion score ${{\varvec{p}}}{\prime}$ for node $i$ is given by

8 $$p_{i}^{\prime } = \frac{{p_{i} }}{{s_{i} }}$$

for unadjusted diffusion score ${p}_{i}$ from RWR and stationary distribution probability ${s}_{i}$. This favors nodes with smaller stationary distribution probabilities, and hence, nodes with smaller degree.

BS and IN are two novel degree bias adjustment methods that act directly on the transition matrix of a random walk, rather than by adjusting diffusion scores post-hoc. Both attempt to manipulate the transition matrix such that the entropy of its stationary distribution is maximized. Maximizing the entropy of the stationary distribution–which is a function of degree–homogenizes the influence of degree on diffusion scores. Bistochastic Scaling, as the name suggests, scales the transition matrix to be approximately bistochastic, which is a matrix with row and column sums all equal to one and possesses the property of having a principal eigenvector with maximum entropy [18]. The scaled matrix can now be used in any network diffusion method relying on a transition matrix, such as RWR. By scaling the transition matrix to be approximately bistochastic, BS increases the entropy of the stationary distribution, thereby mitigating degree influence on diffusion scores.

IN relies on different mechanisms to achieve the same goal of BS, namely the maximization of the entropy of the stationary distribution. IN borrows from the inflation operator of the Markov Clustering algorithm [19]. Elements in each row of the transition matrix are raised to a certain positive power, which is a function of the stationary distribution probability associated with that row, and columns are subsequently re-normalized. This increases stationary distribution entropy by displacing incoming transition probabilities away from nodes as a function of their stationary distribution probability. As with BS, the modified matrix resulting from IN can be used in network diffusion methods such as RWR. See Additional File 1 for more details and pseudocode for IN and BS.

In addition to manipulating the transition matrix once it has been constructed, one can modify how the transition matrix is constructed from the adjacency matrix to combat degree bias. Previous studies have introduced target-aware normalization procedures that calculate transition rates as an inverse function of the degrees of the target and source nodes [7, 17, 20]. We introduce a closely-related procedure called penalized degree normalization, which first modifies the adjacency matrix and then is followed by classic degree normalization. Given an adjacency matrix $A=({a}_{ij})$, let $D$ be a diagonal matrix with diagonal element ${d}_{jj}={\sum }_{i}{a}_{ij}$. A modified adjacency matrix, ${A}{\prime}=({a}_{ij}{\prime})$, is first calculated by

9 $$A^{\prime} = D^{ - k} A$$

for penalization factor $k>0$, with the $i{j}^{th}$ element given by

10 $$a_{ij}^{\prime} = \frac{{a_{ij} }}{{d_{ii}^{k} }}$$

Upon column-normalization, we obtain transition matrix $M=({m}_{ij})$ whose $i{j}^{th}$ element is given by

11 $$m_{ij} = \frac{{d_{ii}^{ - k} a_{ij} }}{{\mathop \sum \nolimits_{l} d_{ll}^{ - k} a_{lj} }}$$

Therefore, penalized degree normalization is an instance of a biased random walk which penalizes transitions to nodes as a function of degree, with penalization factor $k$ controlling the strength of this inverse relationship.

These degree bias adjustment methods and normalization procedures are implemented in the AMEND algorithm. In the context of multiplex or heterogeneous graphs, the chosen degree bias adjustment method can be applied to specific components or layers as specified by the user. This capability allows for active module identification while attenuating degree influence on node selection.

### Multiplex layer aggregation

In the AMEND algorithm, it is possible to collapse multiplex components such that the edge set of a user-specified primary layer is preferentially used during the maximum-weight connected subgraph step. First, network diffusion with RWR-MH is run on the complete multiplex and/or heterogeneous network to obtain diffusion scores. Then, prior to the maximum-weight connected subgraph step, the specified multiplex components are ‘collapsed’—as detailed in the subsequent paragraph—and diffusion scores are aggregated by some user-defined summary function (*e.g.*, mean) for common nodes between layers within a multiplex.

Consider a multiplex component with $M$ layers, where layer $l$ has node set ${V}_{l}=\{{v}_{1},\dots ,{v}_{{n}_{l}}\}$ and edge set ${E}_{l}=\{\left({v}_{i},{v}_{j}\right);{v}_{i} and {v}_{j} are connected\}$, and the primary layer is specified to be layer $\alpha$. The pseudocode below describes how to obtain the node and edge sets of the aggregated multiplex component.
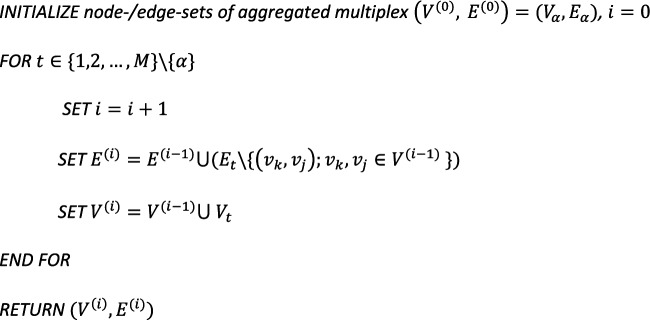


Bipartite edges are preserved for the aggregated components. Upon obtaining a subnetwork from the maximum-weight connected subgraph solution, using the aggregated node and edge sets, the aggregated multiplex components are ‘expanded’ such that it contains the original layers with only nodes from the subnetwork and any edges between them.

### Molecular interaction networks

Various molecular interaction networks were used throughout this study. A complete list of networks, their characteristics, and their uses are given in Additional File 2. The following will describe important pre-processing steps for the construction of multiplex/heterogeneous networks used in the evaluation tasks presented in Results.

For evaluating the ability of RWR-MH to rank features from KEGG pathways, three primary networks were obtained, from which the multiplex/heterogeneous networks were constructed. The multiplex-heterogeneous graph was constructed by linking common proteins between the physical STRING PPI network and the functional *graphite* network, in addition to connecting the MMI network to this multiplex component using bipartite interactions from STITCH.

The TCGA-KIRC analysis involved a multiplex-heterogeneous network of 3 components. The multiplex component, corresponding to the RNA-seq data, contained 4 layers which were connected through common gene-products. The miRNA component, corresponding to the miRNA-seq data, had no miRNA-miRNA interactions; however, miRNA-mRNA interactions from miRTarBase were used to connect the miRNA component to the layers of the RNA-seq component. The third component consisted of genes, to which methylation data from the TCGA-KIRC project were mapped. This methylation component only contained bipartite connections between common gene-products that it shared with the RNA-seq component, as well as between miRNAs of the miRNA component encoded by genes in the methylation component.

The hepatocyte OGT-KO study also involved a multiplex-heterogeneous network containing 4 components corresponding to transcriptomic, proteomic, phospho-proteomic, and metabolomic data. The transcriptomic, proteomic, and phospho-proteomic components each contained two layers: a mouse PPI network of physical and functional interactions from STRING, and a Network of Interactors and Substrates (NISE) for OGT in mouse liver. The STRING layer for each omic type was created by including the 1st order neighbors of the features captured in that omic assay. The NISE layer is a network containing only proteins predicted to be interactors or substrates for OGT in mouse liver [21]. Within each multiplex component, inter-layer edges were created between common nodes. Similarly, the transcriptomic, proteomic, and phospho-proteomic components were connected through common nodes. The metabolomic component consisted of 1-order neighbors of metabolites captured in the metabolomic assay. Bipartite connections from STITCH were applied between the metabolomic component and all of the relevant nodes in the multiplex layers corresponding to the other omic types.

### Experimental data

KEGG pathways, containing both genes and metabolites, were used to assess the effectiveness of RWR-MH in node ranking. Only pathways with at least 5 metabolites and 5 genes were retained, to ensure that each fold contained at least one of each molecular type during fivefold cross-validation. This resulted in 212 pathways. Similarly, Reactome pathways were used to assess the ability to rank nodes for various combinations of degree bias adjustment method and transition matrix type. Only pathways of size 5 or greater were kept, to ensure that each fold contained at least one gene during fivefold cross-validation. This resulted in 1139 pathways.

5 human gene expression datasets were accessed from NCBI GEO (GEO Accession IDs: GSE112680, GSE30219, GSE75214, GSE3790). These were analyzed for differential expression (DE) using the *limma* package [22, 23]. P-values and log fold changes from DE analyses were used as seed values in RWR to compare degree bias adjustment methods and to assess the effectiveness of B-RWR.

Data from the TCGA-KIRC project was accessed through the Broad Institute’s Firehose online interface. mRNA abundances, miRNA abundances, and methylation $\beta$-values (at the gene level) were analyzed for DE between paired tumor and normal tumor-adjacent samples using mixed effects models in *limma*. Exponentiated absolute log fold changes were used as seeds value in the AMEND algorithm for active module identification. Additionally, Gene Ontology and Disease Ontology terms were used in the TCGA-KIRC analysis for functional enrichment analysis of AMEND results.

The OGT-KO study consisted of transcriptomic, proteomic, phospho-proteomic, and metabolomic data from OGT-KO and control samples of mouse liver at 1 and 2 weeks post-KO. DE analysis assessed changes in expression between OGT-KO at 1 week post-KO vs. Control 1 and OGT-KO at 2 weeks post-KO vs. Control 2 to obtain two sets of log fold changes and associated p-values, from which ECIs were calculated for each feature to assess the degree of equivalent or inverse change between the two treatment–control comparisons. ECIs were used as seed values for AMEND, with the algorithm selecting for equivalently changed features between 1 and 2 weeks.

### Functional and disease enrichment

Over-representation analysis (ORA) was used to functionally interpret themodules obtained from the TCGA-KIRC and O-GlcNAc datasets. ORA relies on a hypergeometric test to assess the significance of the overlap between module nodes and features in a pathway. The input network is set as the ‘universe’, and p-values are adjusted for multiple testing using the Benjamini–Hochberg procedure. For the TCGA analysis, a threshold of 0.01 was used to determine statistical significance, whereas a threshold of 0.05 was set for the O-GlcNAc analysis. GO terms representing molecular function, biological process, and cellular compartment were used for the TCGA-KIRC analysis to identify functionality and localization associated with molecules in the module. Additionally, disease ontology (DO) terms were used to assess the modules association with diseases through the DOSE package [24]. KEGG and Reactome pathways were used to assess module functionality for the O-GlcNAc study.

## Results

### Random walk with restart for multiplex/heterogeneous graphs

**Fig. 3:**
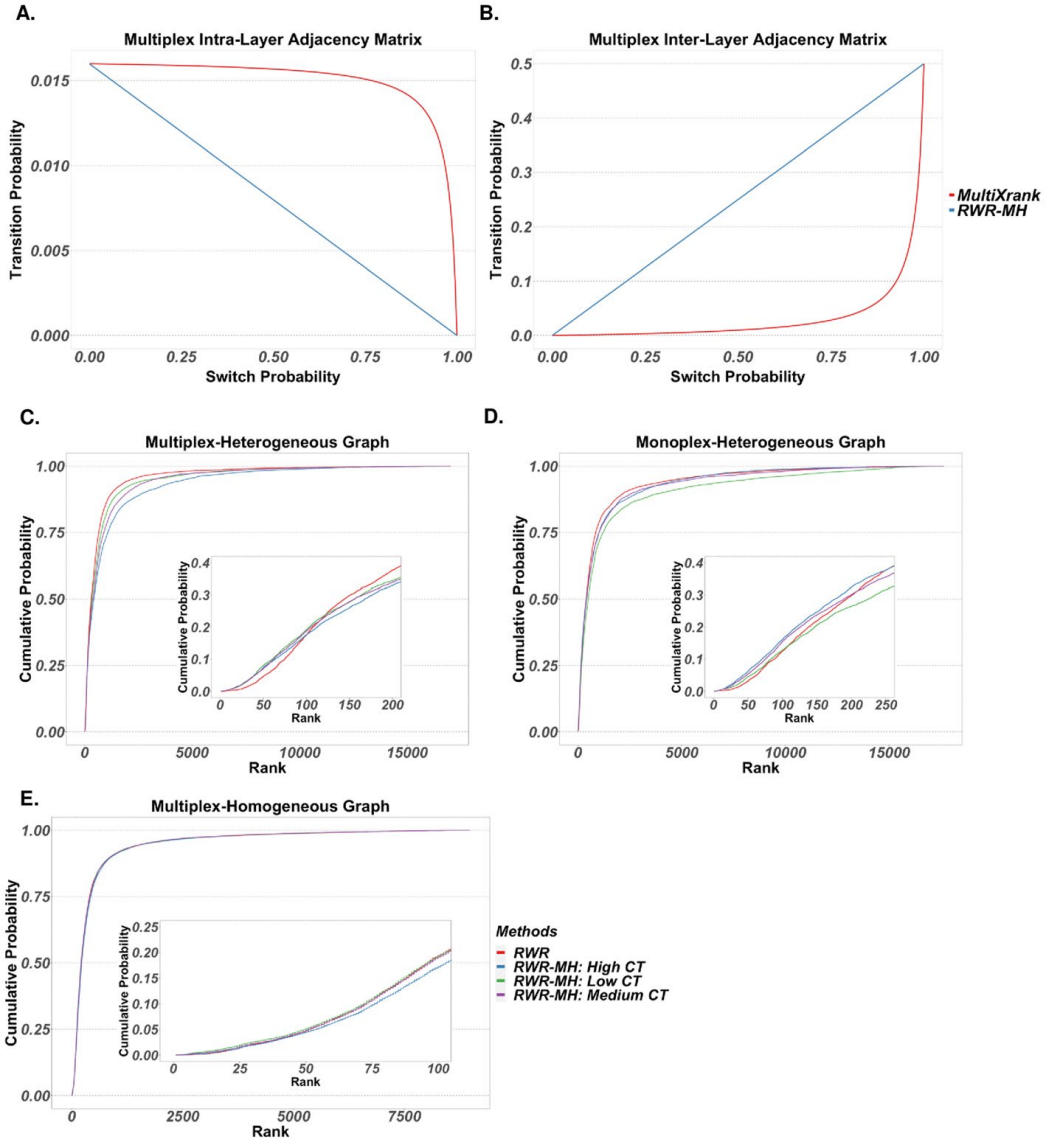
Random Walk with Restart for multiplex/heterogeneous graphs: **A** and **B** Comparison between RWR-MH and MultiXrank for intra- and inter-layer transition probabilities as a function of switch probability, for a hypothetical source-target node pair with source node degree of 50 and source-target edge weight of 0.8 in a multiplex graph with 3 layers. **C**–**E** Empirical cumulative probabilities for ranks of nodes (determined by diffusion scores) in test folds following a fivefold cross-validation procedure using KEGG metabolic pathways, wherein each pathway is split into 5 folds, features in training folds are set as seeds, and the ranks of the features in the test fold are stored. Restart probability is set to 0.5. Ranks shown include ranks from all test folds from all KEGG pathways. Lower ranks represent higher diffusion scores and higher affinity to seed nodes. The networks used include different combinations of three components: a *Mus musculus* physical PPI network from STRING; a functional PPI network wherein an edge connects two nodes if they belong to a common Reactome pathway; and a MMI network of merged isomers from STITCH. The multiplex-heterogeneous graph comprises all three components, with the two PPI networks as layers in a multiplex. The monoplex-heterogeneous graph comprises the functional PPI network and the MMI network. The multiplex-homogeneous graph comprises the two PPI networks as layers

Several authors have previously contributed to the extension of Random Walk with Restart (RWR) to accommodate complex graphs [25–27]. Most recently, MultiXrank was introduced, which generalizes RWR for multiplex and/or heterogeneous graphs [25]. This method can accept graphs with an arbitrary number of node types and edge types and introduces tuning parameters that control the extent of information sharing (cross-talk) between different graph regions during diffusion. While MultiXrank greatly increases the types of graphs to which RWR can be applied, there are several limitations that warrant a reformulation of RWR for multiplex/heterogeneous graphs, which we call RWR-MH. First, MultiXrank requires that each layer within a multiplex component contain the same node set. This greatly limits the types of multiplex graphs it can accept. RWR-MH can accommodate layers with different node sets that may even have zero overlap. The second limitation relates to the normalization of multiplex adjacency matrices (i.e., scaling columns or rows such that they sum to one) during transition matrix construction. A multiplex adjacency matrix comprises inter- and intra-layer adjacency matrices (see *Materials and Methods*). In the MultiXrank formulation, cross-talk parameters are first applied to a multiplex adjacency matrix, which is subsequently normalized as a whole, without distinction between inter- and intra-layer matrices. In RWR-MH, each intra- and inter-layer adjacency matrix is first normalized independently, after which cross-talk parameters are applied. This introduces a re-parameterization that offers more predictable behavior of transition probabilities as a function of cross-talk parameters. This is illustrated in Fig. 3A and B, which show the relationship between the transition and switch probabilities for hypothetical nodes in a multiplex graph. For both intra- and inter-layer transitions, the parameterization in RWR-MH results in a linear relationship between the switch and transition probabilities, which leads to more intuitive behavior compared to the exponential relationship for MultiXrank. Lastly, seed vectors are allowed to differ between multiplex layers in RWR-MH, whereas they must be the same in MultiXrank. This improvement allows for the diffusion of multiple data types in the same multiplex component, which opens up many more possibilities for data integration.

### Feature ranking: RWR-MH versus RWR

RWR-MH was evaluated at the task of ranking nodes that are functionally related to seed nodes. In what follows, lower rank refers to ranks closer to 1 and corresponds to larger diffusion scores. Given the fundamental assumption of guilt-by-association that underlies network diffusion methods, it is expected that a node will be ranked lower if it belongs to common pathways with the seed nodes. Using KEGG metabolic pathways [28], containing both metabolites and genes, we performed a fivefold cross validation procedure, wherein features in the training folds were used as seeds and the ranks of nodes in the test fold were obtained for each pathway. This was performed on 3 graphs: a multiplex-heterogeneous graph with an MMI component and a PPI multiplex component consisting of physical and functional interaction layers (20,653 nodes; 326,459 edges); a monoplex-heterogeneous graph with an MMI component and a PPI component of functional interactions (17,694 nodes; 289,278 edges); and a multiplex-homogeneous graph of a PPI multiplex component consisting of physical and functional interaction layers (12,375 nodes; 198,953 edges). For each graph, RWR-MH was run under three parameter settings representing varying degrees of cross-talk (CT in figures) between layers and components. As cross-talk increases, more information is being shared between components/layers. High, medium, and low cross-talk correspond to values of 0.85, 0.5, and 0.15, respectively, for all cross-talk parameters (denoted as $\lambda$ and $\delta$ in *Materials and Methods*). For this and all subsequent analyses involving RWR (except when run in the AMEND algorithm), the restart probability parameter is set to 0.5.

Figure 3C-E show empirical cumulative probabilities for the ranks of nodes in the hold-out folds. Lines that lie above others at a certain rank represent a larger probability of assigning ranks less than or equal to that value, and thus, an improvement in that methods ability to give low ranks to nodes that are functionally related to seed nodes. For Fig. 3C–D, insets reveal a slightly superior performance from RWR-MH when considering top-ranked nodes. However, Fig. 3C inset highlights the ambiguity of the results. When considering ranks of 100 or lower, RWR-MH out-performs RWR, regardless of cross-talk. But when considering ranks larger than 100, RWR out-performs the others. The impact of cross-talk varies between graphs. For the multiplex-homogeneous graph (Fig. 3E), high cross-talk negatively impacts performance, albeit subtly. For the multiplex-heterogeneous graph (Fig. 3C), we see the worst performance among the RWR-MH methods for the high cross-talk scenario, whereas high cross-talk shows the best performance for the monoplex-heterogeneous graph (Fig. 3D). This suggests that the appropriate amount of cross-talk between graph components depends on the graph topology. For this analysis, large cross-talk for multiplex graphs results in poorer performance, possibly due to the reduced ability of the random walker to thoroughly explore each layer individually.

### Degree bias adjustment

PPI networks have been shown to suffer from degree bias, wherein certain proteins may have an inflated or deflated number of interactors (i.e., node degree) relative to others [29]. This is the result of various technical and study biases that are inherent in PPI ascertainment studies; for example, certain technologies may favor the detection of interactors for highly-abundant proteins, while some proteins are simply studied more often due to their association with high-profile diseases [29]. This presents problems for network diffusion methods, which partly determine node importance based on degree. Several approaches for addressing these issues in the context of network diffusion and active module identification (AMI) methods have been put forward. These range from diffusion score adjustments to permutation-based approaches, such as permuting seed vectors or diffusing on random degree-preserving networks (RDPN) to obtain empirical p-values for diffusion scores [7, 17, 30]. In the Discussion, we’ll present reasons why RDPN-based approaches may be misguided. But in the context of AMEND, which is an iterative algorithm that runs RWR on many different (sub)networks, permutation-based approaches are too computationally intensive. We choose to pursue an unexplored mode of degree bias adjustment by directly adjusting the transition matrix, post-normalization but prior to diffusion. We also investigate the effects of different adjacency matrix normalization methods for constructing the transition matrix.

We compare three different degree bias adjustment methods: stationary distribution scaling (SDS), inflation-normalization (IN), and bistochastic scaling (BS). SDS, also called eigenvector centrality scaling and previously introduce by Erten et al. (2011), involves scaling diffusion scores by stationary distribution probabilities. The stationary distribution is the normalized eigenvector associated with the absolute largest eigenvalue of the transition matrix. Both IN and BS are novel adjustment methods being introduced here that involve manipulating the transition matrix prior to diffusion. Both are based on the assumption that the stationary distribution of a transition matrix is a fair metric for assessing individual influences of node degree on diffusion scores. They attempt to maximize the entropy of this distribution, thereby squeezing degree influence of all nodes towards a global mean. IN applies exponents to the rows of a left-stochastic transition matrix, followed by column re-normalization. BS implements Iterative Proportional Fitting (IPF) to scale the transition matrix to be approximately bistochastic [31]. Additionally, we compared two transition matrices obtained from different adjacency matrix normalization procedures: degree normalization and penalized degree normalization with varying penalization factors (see *Materials and Methods*). These methods were compared in three evaluation tasks: feature ranking, assessment of correlation between diffusion scores and node degree, and subnetwork identification in the context of the AMEND algorithm.

**Fig. 4:**
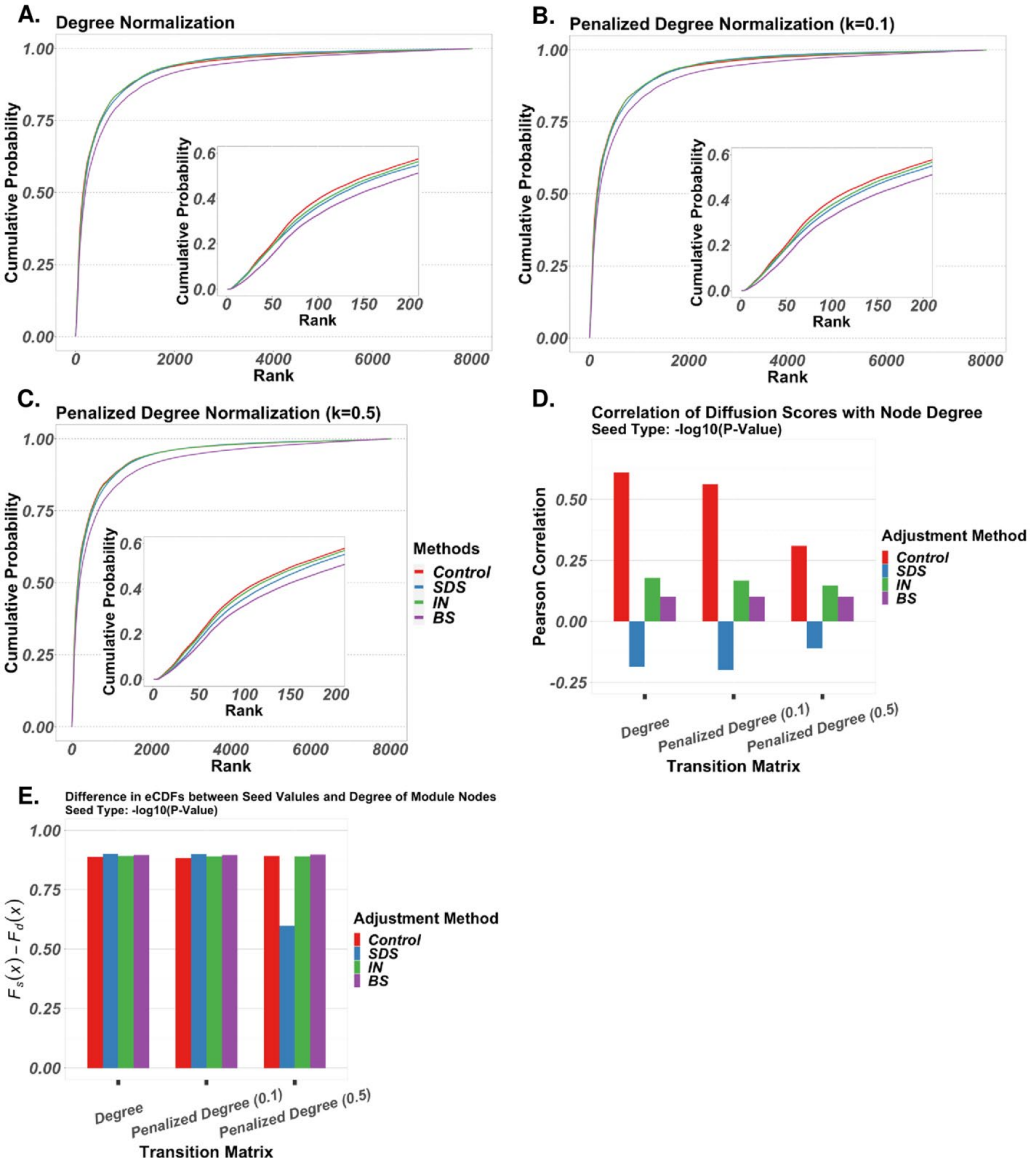
Degree bias adjustment analysis: combinations of transition matrix types and degree bias adjustment methods compared on 3 tasks: ranking of functionally related proteins, correlation between diffusion scores and degree, and retention of high-seed-value nodes in the context of AMEND. For all figures, the restart probability is set to 0.5 for RWR. **A–C** Empirical cumulative probabilities of ranks. Using Reactome *Mus musculus* pathways and a *Mus musculus* PPI network of physical and functional interactions, a fivefold cross validation procedure was implemented in which pathways are split into folds, nodes belonging to the training folds of a pathway are used as seeds in RWR, and the ranks of diffusion scores of nodes in the test fold are collected (higher score equals lower rank). Ranks shown in figures include ranks from all test folds from all pathways. **D** Correlation between diffusion scores and node degree. For each of 5 human gene expression datasets, -log_10_-transformed p-values from differential expression analysis were diffused on a human PPI network of functional and physical interactions. Pearson correlation coefficients were averaged across datasets. **E** Average difference in empirical cumulative probabilities between seed values and degree for module nodes returned from AMEND. For each of 5 human gene expression datasets, seed values are -log_10_-transformed p-values from differential expression analyses assigned to nodes such that seed value and degree are perfectly negatively correlated. Results are averaged across datasets

The first task involves a fivefold cross-validation procedure analogous to the previous section, wherein genes from Reactome *Mus musculus* pathways [32] in the training folds are used as seeds, diffusion is run on a *Mus musculus* physical PPI network from STRING [33], and the ranks of diffusion scores for nodes in the test fold are assessed (higher scores equals lower ranks). Figure 4A–C show cumulative probabilities for ranks of genes in hold-out folds for combinations of transition matrix and adjustment method. For all three transition matrix types, the control method of no adjustment results in the best performance, as shown by the insets. IN performs slightly better than SDS and BS, with BS showing the worst performance across all three transition matrix types. Performance is similar across the transition matrix types.

Next, correlation between diffusion scores and degree was assessed using 5 human gene expression datasets obtained from NCBI GEO [34] that were diffused on a human PPI network of physical and functional interactions from STRING. Results were averaged across datasets. Figure 4D, obtained using -log_10_ transformed p-values as seeds, shows marked differences in correlation among the adjustment methods. As expected, the control results in the largest Pearson correlation coefficient between diffusion score and degree. We also see correlations closer to zero for penalized degree transition matrices, which is expected since this biases outgoing transition probabilities towards low-degree nodes. Interestingly, BS shows little variation in correlation between transition matrix types, and SDS results in negative correlation between diffusion score and degree, which is undesirable given that node degree, despite being a corrupt metric, still offers evidence of biological importance [35]. IN also shows a large decrease in correlation. Similar results are obtained using absolute log fold changes as seeds (Additional File 3).

The goal of the final degree bias analysis was to assess the extent to which AMEND retains nodes based on seed value compared to degree. Transition matrix types and adjustment methods were implemented in the AMEND algorithm and run using the 5 gene expression datasets and the PPI network introduced previously, with results being averaged across datasets. To disentangle the effects of seed value and degree on node retention in the final module, seed values were assigned to nodes such that degree and seed value were perfectly negatively correlated. For each module returned from AMEND, the average difference in empirical cumulative probabilities between the seed values and degrees of module nodes was calculated with respect to the entire network. To the extent that AMEND learns primarily from seed values and not from degree, we would expect these differences to be close to 1. Figure 4E, obtained using -log_10_ transformed p-values as seeds, shows fairly uniform, positive results across the transition matrices and adjustment methods, with a notable exception being SDS for penalized degree normalization ($k=0.5$). For the degree and penalized degree ($k=0.1$) transition matrices, SDS shows the largest difference, followed by BS, then IN, and then control. This corroborates Fig. 4D, which shows increasing correlation between diffusion score and degree when moving from SDS to BS to IN to control. The large differences in empirical cumulative probability between seed value and degree indicate that AMEND accurately retains nodes with large seed values, despite these nodes having low degree. Results are slightly more variable when using absolute log fold change as seed values, although all y-axis values remain above 0.75 (Additional File 3).

Despite performing worse than control in the feature ranking analysis, the IN adjustment method outperformed SDS and BS. Also, IN showed a significant decrease from the control in correlation between diffusion score and degree, while remaining positive. It also showed favorable results in its ability to retain high-seed-value, low-degree nodes in the context of AMEND. Given these trade-offs, IN is recommended as the preferred degree bias adjustment method and is employed for the analysis of the real-world datasets included in this study.

### Biased random walk

In its broadest sense, a biased random walk (BRW) is any random walk in which there are non-uniform transition probabilities from a node to its neighbors. For our purposes, the definition of a biased random walk is restricted to be a random walk in which node-wise, non-negative values are applied in such a way that nodes with larger values have increased incoming transition probabilities (see *Materials and Methods*). We will use the term ‘BRW attribute’ to refer to these non-negative values. Biased Random Walk with Restart (B-RWR) can be used to select for nodes that have large seed values and large BRW attribute values, effectively transforming RWR into a multi-objective algorithm. B-RWR has been implemented into the AMEND algorithm.

**Fig. 5:**
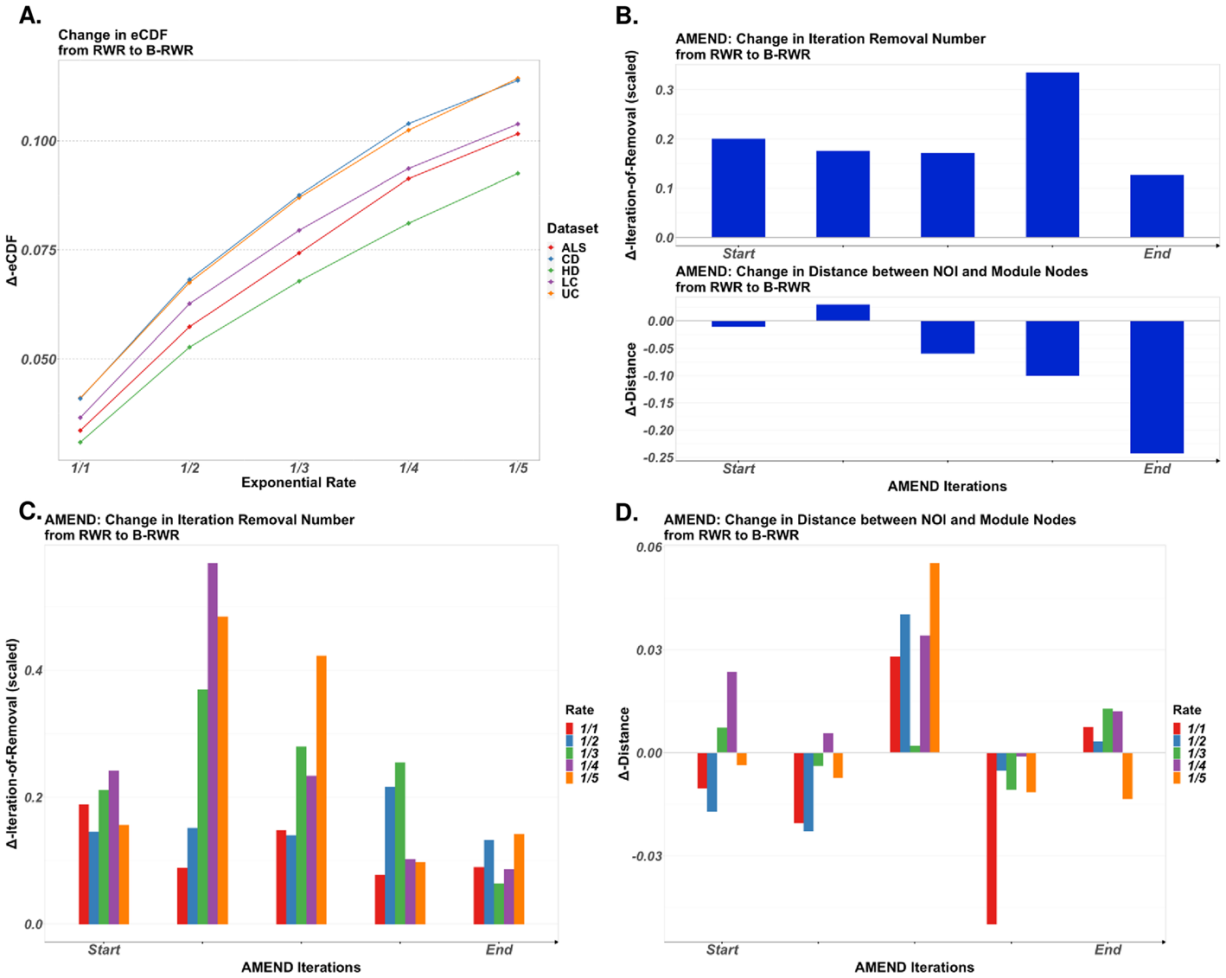
Biased random walk: impact of biased random walk with restart (B-RWR) on diffusion scores and on AMEND modules. Seed values are −log_10_(*p*-value), with p-values coming from differential expression analysis of 5 human gene expression datasets. For panels **B–D**, results are averaged across datasets. In the context of AMEND, nodes of interest (NOI), chosen to be nodes that were removed at varying points in the iterative AMEND algorithm, were assigned large BRW attribute values. **A** 10% of nodes in the PPI network, chosen randomly, were assigned BRW attribute values from an exponential distribution with varying rate parameter, while all other nodes were set to 1. Y-axis shows the change in empirical cumulative probabilities for diffusion scores of these selected nodes between RWR and B-RWR, for 5 gene expression datasets. Restart parameter is set to 0.5. **B** B-RWR in the AMEND algorithm. All nodes were assigned BRW attribute values that decrease as a function of distance to NOI in the PPI network. Upper plot shows the change in average iteration-of-removal of NOI from AMEND_RWR_ to AMEND_B-RWR_. Lower plot shows the change in average distance (in full PPI network) between module nodes and NOI from AMEND_RWR_ to AMEND_B-RWR_. **C–D** Similar to panel **B**. The BRW attribute values assigned to NOI are drawn from an exponential distribution with varying rate parameters, with all other nodes set to 1

Figure 5A–D show the impact of B-RWR on raw diffusion scores and also in the context of AMEND. For analyses in this section, seed values were set to be −log_10_(*p*-value) coming from differential expression analyses of 5 human gene expression datasets (see *Materials and Methods*). For Fig. 5B-D, results were averaged across datasets. For Fig. 5A, 10% of nodes in a PPI network were chosen at random and assigned BRW attribute values from an exponential distribution with varying rate parameters, while all other nodes were given a value of 1. Empirical cumulative probabilities of diffusion scores for these selected nodes were compared between RWR and B-RWR, and Fig. 5A shows a marked increase from RWR to B-RWR, with this increase becoming greater as the rate parameter approaches 0. As expected, B-RWR increases diffusion scores for nodes with large BRW attribute values.

Figure 5B–D capture the impact of B-RWR when embedded in the AMEND algorithm, which by default relies on RWR to assess node importance. This impact was assessed in two ways. First, given the iterative nature of AMEND, we compared the iteration at which selected nodes of interest (NOI) were removed from the network between AMEND with RWR (AMEND_RWR_) and AMEND with B-RWR (AMEND_B-RWR_); it is expected that this will increase in AMEND_B-RWR_. Second, we captured the distance (in the full PPI network) from module nodes (i.e., nodes in the final module returned from AMEND) to NOI; it is expected that AMEND_B-RWR_ will reduce the distance between module nodes and NOI. These NOI were chosen to be nodes that were removed at certain iterations in the AMEND algorithm, ranging from early iterations (these nodes are considered to be less important) to later iterations (these nodes are considered to be more important). For Fig. 5B, BRW attribute values were set such that they decrease as a function of distance from NOI (see *Materials and Methods*). There is a steady increase in iteration removal number (scaled by the total number of iterations in that run), which means that these nodes are staying in the network longer and thus have more influence on the diffusion scores of other nodes. We also see a slight decrease in distance between module nodes and NOI when we compare AMEND_RWR_ to AMEND_B-RWR_. For Fig. 5C–D, NOI are being assigned BRW attribute values drawn from an exponential distribution with varying rate parameters, while values of 1 are given to all other nodes. Figure 5C shows a clear increase in iteration removal number. The change in distance, given in Fig. 5D, gives more ambiguous results. Given the very small scales (y-axis ranges from − 0.03 to 0.06), we can conclude that AMEND_B-RWR_, when assigning BRW attribute values from an exponential distribution, doesn’t have a substantial impact on the final module, in terms of distance to NOI. However, the impact is more substantial when assigning BRW attribute values that decrease as a function of distance from NOI (Fig. 5B).

### AMEND 2.0: new capabilities

The methods introduced in the previous sections—RWR-MH, B-RWR, degree bias adjustment methods, and adjacency matrix normalization methods—have been implemented into the previously introduced AMEND algorithm for active module identification. RWR-MH enables the analysis of multi-omic data in a multiplex, heterogeneous network with an arbitrary number of components and layers. The biased random walk capability enables the integration of secondary, node-wise information that informs node selection in addition to seed values. Furthermore, users have the option to implement degree bias adjustment in a component/layer-specific fashion. Additionally, in the context of multiplex networks, it is possible to collapse multiplex components such that the edge set of a user-specified layer is preferentially used during the maximum-weight connected subgraph step of the algorithm (see *Materials and Methods: Multiplex Layer Aggregation*). Together, these new capabilities address important needs in the area of active module identification, and they make AMEND a flexible tool that facilitates multi-omic data integration and knowledge discovery in biomedical research. The AMEND algorithm is available as a package in the R software environment [36], with installation instructions and tutorials at https://github.com/samboyd0/AMEND. General guidelines on data preparation and function argument specification can be found in Additional File 1.

### AMEND identifies key molecular features involved in kidney renal cell carcinoma

We applied the AMEND algorithm to two real-world datasets: the TCGA-KIRC study and an O-GlcNAc perturbation study of mouse liver. The Cancer Genome Atlas (TCGA) was a pan-cancer initiative that molecularly profiled over 20,000 tumor and paired-normal samples for 33 cancer types [37]. For this analysis, RNA-seq, miRNA-seq, methylation, and clinical data from the TCGA Kidney Renal Cell Carcinoma (TCGA-KIRC) project were obtained through the Broad Institute’s Firehose [38]. The RNA-seq, miRNA-seq, and methylation datasets had 72, 68, and 160 paired tumor-normal samples, respectively. Differential expression analysis between paired tumor and normal samples yielded log fold changes for each omic type, with exponentiated absolute log fold changes serving as seed values. Hence, AMEND searched for both up- and down-regulated features. Additionally, hazard ratios of mortality were obtained from Cox proportional hazard models for each feature in the omics data. These values were mapped to nodes in a multiplex-heterogeneous network that comprised three components corresponding to RNA-seq, miRNA-seq and methylation data. The mRNA component is multiplex with four layers: a human PPI network of physical interactions from STRING; a kidney-specific human PPI network from OhmNet [39]; a gene regulatory network constructed from TCGA-KIRC gene expression data using ARACNE [40]; and a network from the *graphite* package [41] comprising edges between genes that lie in common Reactome pathways. The miRNA component contained only bipartite connections between miRNAs and mRNAs, using interaction information from the miRTarBase database [42]. The methylation component, where methylation values are summarized at the gene level, consist of genes captured in methylation data, with edges connecting common genes between mRNA-methylation components and between miRNA-encoding genes and their miRNA products in the miRNA component. This network contains 57,642 nodes and 747,549 edges.

**Fig. 6:**
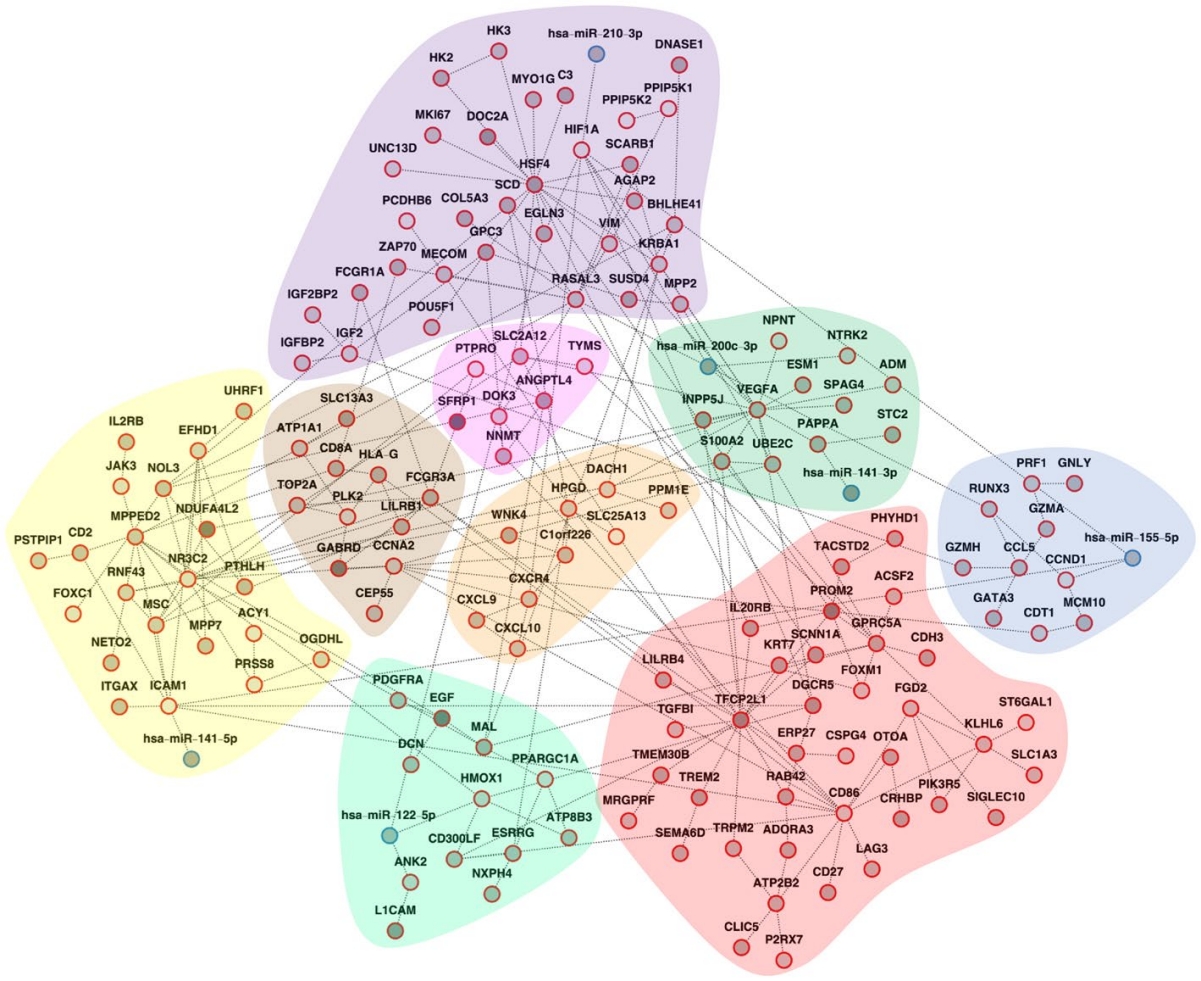
Active module from TCGA-KIRC analysis with AMEND: AMEND module using TCGA-KIRC multi-omic data. Darker shades of grey for node color represent larger seed values, which were exponentiated absolute log fold changes from differential expression analyses. Hazard ratios were set as BRW attribute values. Red node borders represent the RNA-seq component, while blue node borders represent the miRNA-seq component. The multiplex RNA-seq component was collapsed such that the STRING physical PPIs are preferentially considered during the maximum-weight connected subgraph step of the AMEND algorithm

Figure 6 shows the AMEND module after diffusing RNA-seq, miRNA-seq, and methylation data simultaneously in a multiplex-heterogeneous network. Furthermore, hazard ratios were supplied as BRW attribute values to select for features whose expression correlates with mortality. IN was also applied to the mRNA component to mitigate degree bias in the PPI networks. There are six miRNAs in the module, all of which have clear connections with renal cell carcinoma (RCC). miR-122, miR-155, and miR-210, all up-regulated in the TCGA-KIRC data, have shown significant up-regulation in RCC samples, promoting tumor progression, proliferation, and migration [43, 44]. miR-200c and miR-141, both of which are down-regulated in the TCGA-KIRC data, have shown significant down-regulation in RCC samples, contributing to cell proliferation and metastasis [45, 46]. The module contains *VEGFA*, an oncogene whose gain of function is due to under-expression of miR-200c and miR-141 [47]. The module also contains *HIF1A*, a subunit of hypoxia-inducible factor 1 which, due to its dysregulation in RCC cells, promotes the expression of miR-210 [48]. Several genes implicated in RCC were also captured in the module, including *TGFBI* [49], *ICAM1* [50], and *VIM* [51].

**Fig. 7:**
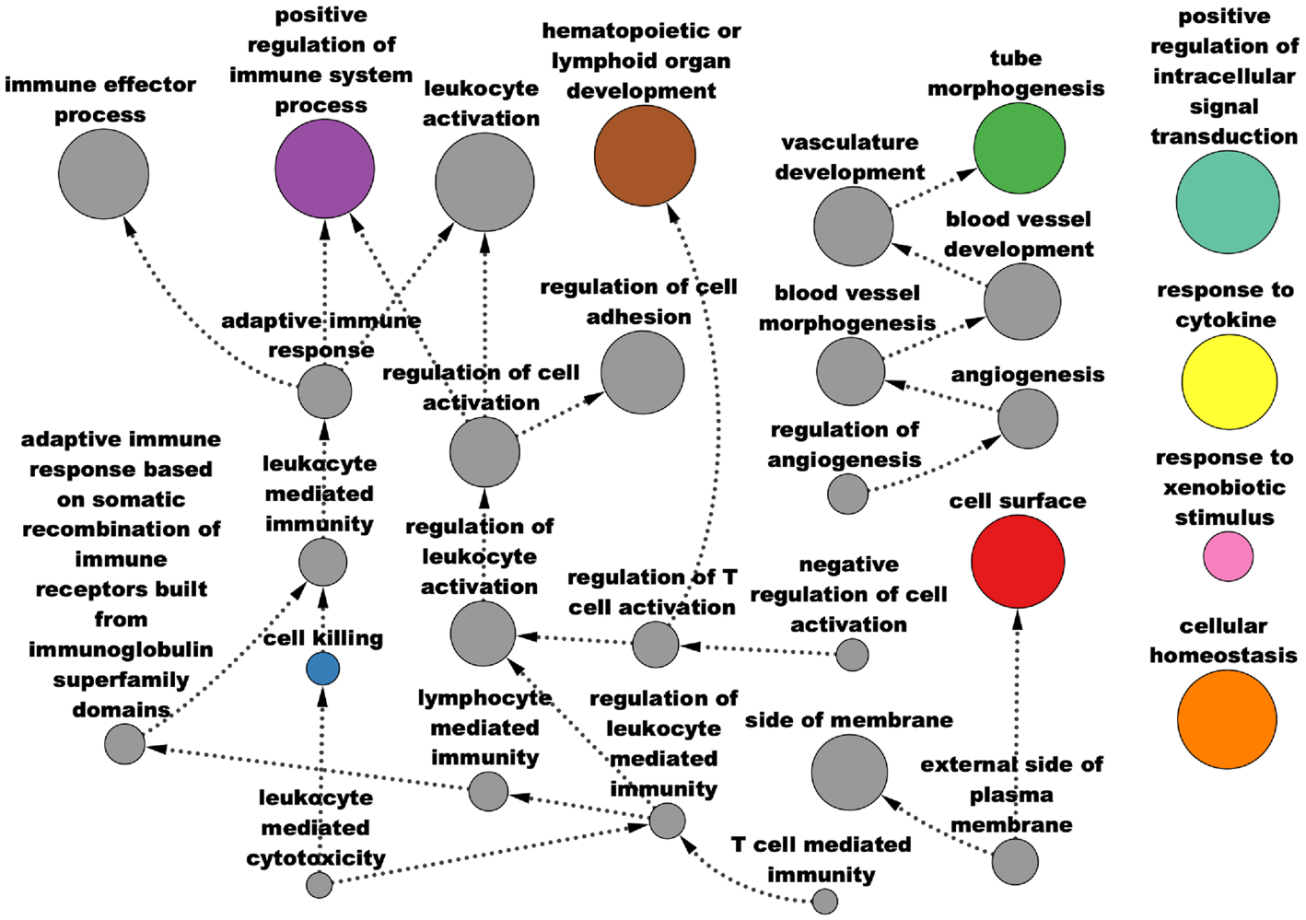
Pathway analysis of AMEND module from TCGA-KIRC data: significant GO terms from over-representation analysis of the AMEND module from TCGA-KIRC data, using a BH-adjusted p-value cutoff of 0.01, are displayed as an acyclic, directed graph depicting terms nested within others. Colors correspond to clusters in the AMEND module that contain the most features of that term. Node size corresponds to the number of features in that GO term

Over-representation analysis (ORA) of GO terms [52, 53] also picks up important pathways that are characteristic of cancer (Fig. 7, Additional File 4). The green cluster—containing *VEGFA*, miR-200c-3p, and miR-141-3p—is associated with angiogenesis, with dysregulated angiogenesis being a hallmark of cancer [54]. The purple cluster—containing miR-210-3p, *HIF1A*, and *VIM*—is associated with positive regulation of immune system processes and adaptive immune response. Another relevant biological process significantly associated with the module is the regulation of T cell activation, relating to genes such as *CD2*, *CD86*, and *JAK3*; T cells play an important role in the tumor microenvironment (TME) and have been shown to make up the majority of immune cells in the TME of RCC [55]. Furthermore, disease enrichment with Disease Ontology terms [56] show significant enrichment for many cancer-related conditions, including RCC (Additional File 5).

**Fig. 8:**
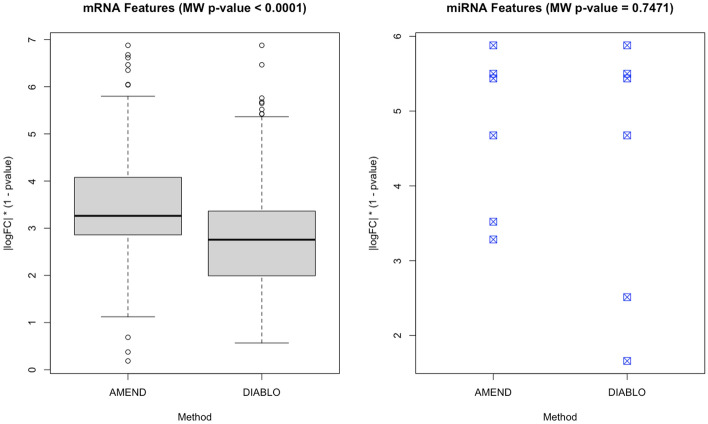
AMEND-DIABLO comparison on TCGA-KIRC data: comparison of log fold change distributions for feature sets returned by AMEND and DIABLO. For both methods, the mRNA and miRNA feature set sizes are 150 and 6, respectively. A non-parametric Mann–Whitney U test was performed to test for differences in the distribution of weighted log fold changes between the two methods

While there are few, if any, network-based methods that can flexibly integrate multi-omic data for the purpose of module identification/feature selection, there are other, non-network-based methods that achieve a similar purpose. Among them is DIABLO, a supervised multi-omic integration and feature selection method based on maximizing covariance between input datasets [57]. DIABLO was applied to the TCGA-KIRC data to obtain a feature set from the mRNA-seq, miRNA-seq, and methylation datasets that also differentiates between normal vs. tumor samples. A non-parametric Mann–Whitney U test was performed to test for differences in distribution of log fold changes (weighted by 1 minus p-value) between the feature sets returned by AMEND and DIABLO. The weighted log fold changes of the mRNA features from the AMEND module (N = 150) were significantly higher than those from the first factor given by DIABLO (N = 150), despite a moderate overlap (Jaccard Index of 0.31) (Fig. 8). The distributions of weighted log fold changes for the miRNA features were not significantly different between AMEND (N = 6) and DIABLO (N = 6), partly due to the large overlap between them (Jaccard Index of 0.67).

Additionally, AMEND provides the feature set in the context of an interaction network, making it possible to explicitly define intra- and inter-omic relationships between molecules based on a priori functional and physical interaction data. The module from AMEND is also amenable to community detection algorithms to identify clusters of molecules in the module that may be functionally related. DIABLO requires the same samples to be present across all datasets. While this scenario is ideal in a multi-omic experiment, often it is not the case. These conditions do not hold for the next example; therefore, DIABLO could not be used for comparison.

### OGT knockdown disrupts mitochondrial and peroxisomal lipid metabolism

We next applied the AMEND algorithm to a study of O-GlcNAc alterations in mouse liver. O-GlcNAc is a ubiquitous post-translational modification (PTM) of serine and threonine residues of intracellular proteins. This PTM regulates many essential biological functions such as gene transcription, protein translation, and signal transduction [58, 59]. O-GlcNAc is mediated by two enzymes: O-GlcNAc transferase (OGT), which adds O-GlcNAc to target proteins, and O-GlcNAcase (OGA), which removes O-GlcNAc from target proteins. Our data consist of transcriptomic, proteomic, phospho-proteomic, and metabolomic data from wildtype and OGT knockdown (KO) mouse liver at 1 or 2 weeks post-KO. To identify stable effects of OGT-KO, we calculated the degree of equivalent change between OGT-KO vs. control at 1 week and OGT-KO vs. control at 2 weeks using the equivalent change index (ECI) [60, 61]. ECIs are used as seed values in the AMEND algorithm. The molecular interaction network comprises 4 components corresponding to the 4 omics types. The transcriptomic, proteomic, and phospho-proteomic components are multiplex with 2 layers each: a mouse PPI network of functional interactions from STRING and a network of interactors and substrates of OGT in mouse liver (see *Materials and Methods*). The metabolite interactions come from STITCH [62], as well as the bipartite edges connecting the metabolomic component to the others. The transcriptomic, proteomic, and phospho-proteomic components are linked through common gene-products. This network contains 33,504 nodes and 710,147 edges.

**Fig. 9:**
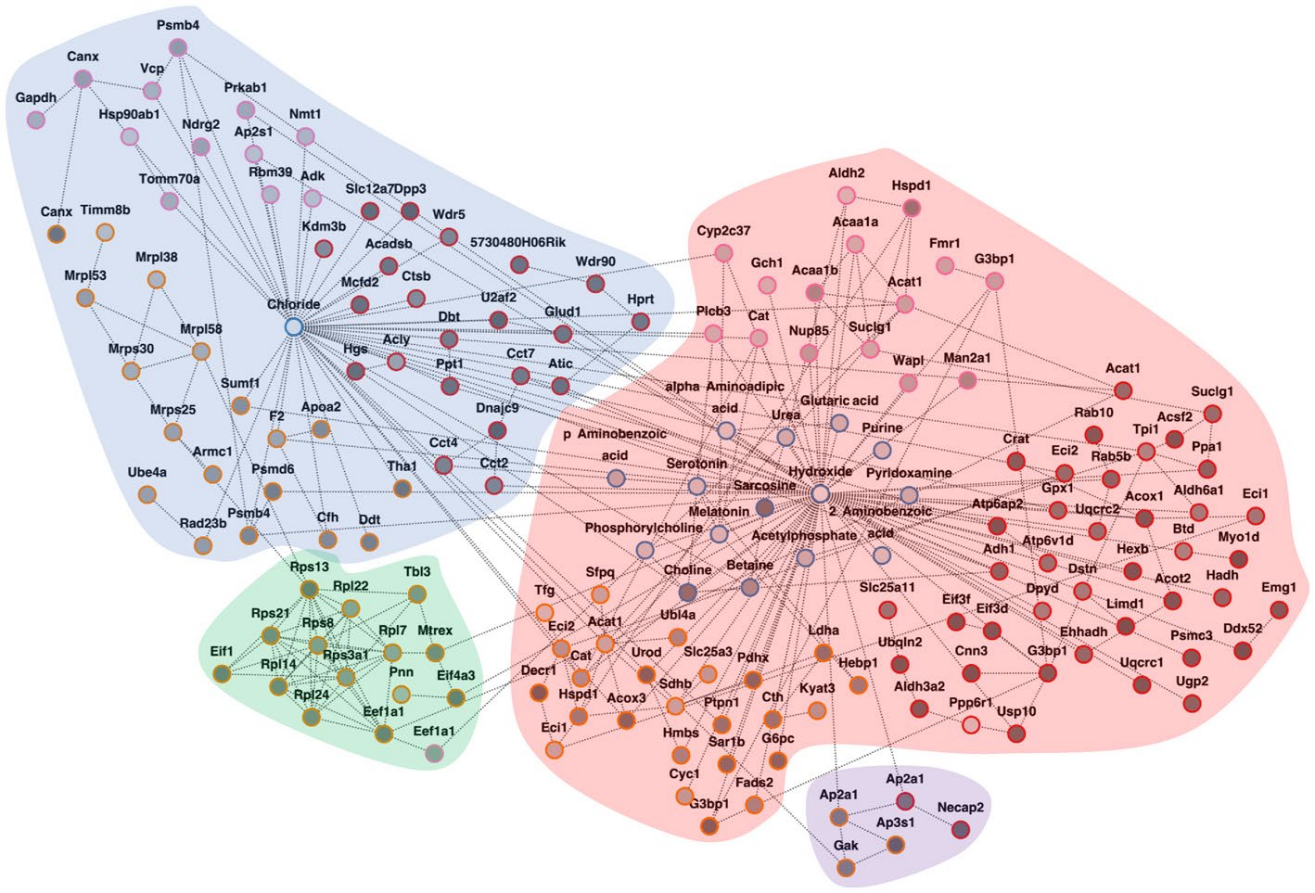
O-GlcNAc analysis with AMEND: active module obtained from AMEND. Seed values for AMEND are equivalent change indices (ECIs), which are weighted ratios of log fold changes between OGT knockout vs. Control at 1 week and OGT knockout versus control at 2 weeks from differential expression analyses of transcriptomic, proteomic, phospho-proteomic, and metabolomic data. Samples come from mouse liver. Darker grey nodes represent nodes with ECIs closer to 1 (more equivalently changed between treatment–control comparisons). Red, orange, pink, and blue node borders correspond to the transcriptomic, proteomic, phospho-proteomic, and metabolomic components, respectively

**Fig. 10:**
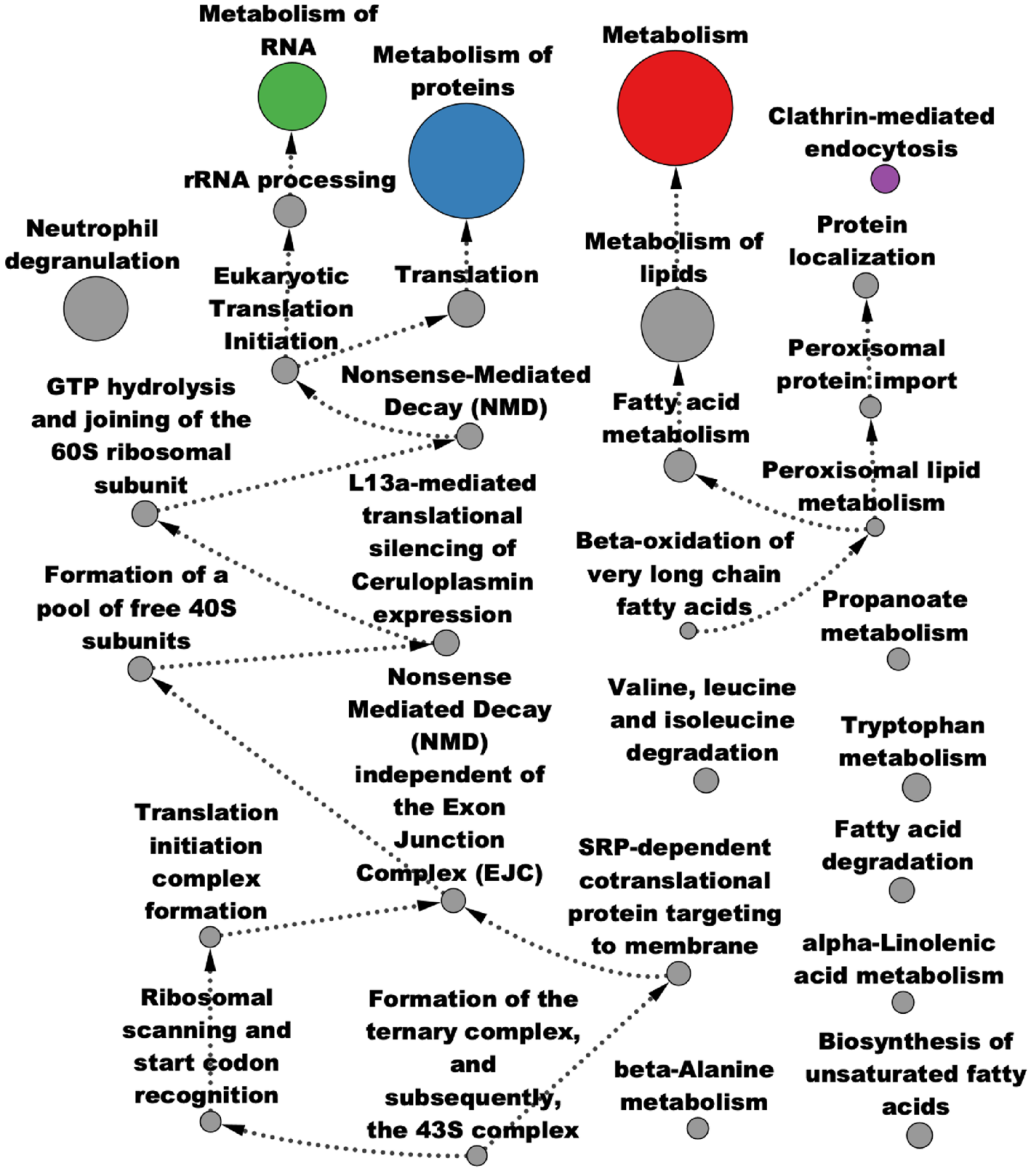
Pathway analysis of AMEND module from O-GlcNAc data: significant pathways from over-representation analysis (ORA) of the AMEND module obtained from the O-GlcNAc dataset. A directed, acyclic graph of significant Reactome and KEGG pathways from ORA, with a BH-adjusted p-value cutoff of 0.05. Edges point from smaller pathways to larger pathways in which they are partially nested. Colors correspond to clusters in the AMEND module that contain the most features of that pathway. Node size corresponds to pathway size

The AMEND module (Fig. 9) contains 167 nodes (63 mRNAs, 60 proteins, 28 phospho-proteins, 16 metabolites) and 290 edges. In what follows, nodes coming from the transcriptomic, proteomic, and phospho-proteomic components of the network will be denoted by T, P, and Ph, respectively. The colored clusters correspond primarily to metabolic processes. Among nodes in the red cluster corresponding to metabolism, there is Choline, an essential nutrient in lipid metabolism with links to O-GlcNAc [63, 64], which was down-regulated in OGT-KO samples at both 1 and 2-weeks post-KO. The module also contains metabolites of Choline: Betaine and Sarcosine. While Sarcosine was also down-regulated at both timepoints, Betaine showed consistent up-regulation at 1 and 2-weeks post-KO. These metabolites, along with proteins *THA1* (P) and *CTH* (P) captured in the module, belong to the Glycine, Serine, and Threonine Metabolism pathway from KEGG (Additional File 6). Additionally, we see several genes, gene products, and pathways related to lipid metabolism in both mitochondria and peroxisomes (Figs. 9 and 10). *DECR1* (P) and *ACSF2* (T), both involved in fatty acid beta-oxidation in mitochondria, have previously been found to be down-regulated after O-GlcNAcylation impairment [65], which is corroborated by our data. Additional enzymes involved in mitochondrial fatty acid beta-oxidation are *ECI1* (T,P) and *ECI2* (T,P). These enzymes show equivalent down-regulation across omic datasets in OGT-KO samples between 1 and 2-weeks, with the notable exception of *ECI2* with consistent up-regulation in transcriptomics but down-regulation in proteomics across 1 and 2 weeks post-KO. Gene products captured in the module that relate to peroxisomal lipid metabolism include *ACOX1 (*T*), ACOX3 (*P*), ALDH3A2 (*T*), CRAT (*T*), ACAAB1 (*Ph*),* and *EHHADH (*T*)*, most of which show equivalent down-regulation across 1 and 2-weeks post-KO, excluding *CRAT* (T) and *ACOX3* (P). These findings suggest that O-GlcNAcylation is a novel regulator of lipid metabolism in mouse liver.

## Discussion

We present a new version of the AMEND algorithm, which now allows for multi-omic data integration and active module identification on multiplex-heterogeneous networks using the RWR-MH network diffusion method, with the options of biased random walk for multi-objective node selection (B-RWR) and degree bias adjustment. We assessed the effectiveness of RWR-MH, B-RWR, degree bias adjustment methods, and transition matrix types on various tasks such as feature ranking, degree bias mitigation, and module identification. These individual methods were wrapped in the AMEND algorithm and applied to two diverse multi-omic datasets coming from the TCGA-KIRC project and an OGT knockout study. While most existing methods have been developed for specific data types and research questions, thereby limiting their generalizability, AMEND is designed to be a flexible tool that can accommodate many different combinations of multi-omic data and network configurations.

AMEND is robust to the dimensions of the input datasets. Small sample sizes will affect omics analyses that are performed as a pre-processing step for AMEND; however, since AMEND can accept any node-wise metric derived from the experimental data, sample size considerations should be specific to the type of analysis being performed (e.g., differential expression analysis). In addition, the omics experiment does not need to contain all of the molecules in the network used for AMEND. Nodes corresponding to molecules not captured in the assay will be given a seed value of 0 but are nonetheless included in the analysis, with the possibility of being included in the final module by virtue of network diffusion.

RWR-MH is a reparameterization of MultiXrank that leads to more intuitive behavior over the entire parameter space. It also introduces some important generalizations beyond MultiXrank that enable RWR-MH to accommodate multiplex layers with varying node sets and seed vectors. RWR-MH improves over RWR in the task of node ranking when considering top-ranked node, which is the most relevant since one of the purposes of ranking is to reduce the feature-space to a more manageable and interpretable subset. Although RWR-MH may perform as well as or worse than RWR in some scenarios (e.g., multiplex-homogeneous), it offers capacities such as cross-talk and seed weight control which are advantageous in other cases, such as when a specific omic type of a multi-omic experiment is of more interest than others. A limitation of this analysis is that cross-talk values are uniform across all layers/components, rather than being tuned to optimality for the specific network/data configuration. This was done to more easily assess the impact of cross-talk on node ranking. The limited impact of RWR-MH for the multiplex graphs could be due to the limited extra information that each layer offers, since STRING borrows information from pathway databases when curating their PPIs [66].

Two novel degree-bias adjustment methods were introduced–BS and IN– that directly manipulate the transition matrix prior to diffusion to attenuate the influence of degree on diffusion scores. IN had the best performance when considering its ability to rank nodes, to decrease correlation between diffusion scores and degree, and to select low-degree, high-seed-value nodes in the context of AMEND. While all adjustment methods showed poorer performance compared to control in their ability to rank proteins associated with seed proteins, this may be due to the possibility that many proteins in popular pathway databases (e.g., Reactome) suffer to some extent from study bias, and consequently, from degree bias in the context of a PPI network. Degree bias adjustment may be most appropriate in scenarios where underexplored proteins are desired.

Biased random walk with restart (B-RWR) was shown to effectively increase the rank of nodes based on a continuous attribute. In the context of AMEND, the impact of B-RWR is limited, although it does cause nodes to stay in the network longer, thereby increasing their influence on other nodes. The penalized degree normalization method is an instance of a biased random walk, with inverse degree serving as the node attribute. This illustrates the versatility of B-RWR, which can be used not only to increase transition rates to desirable nodes, but conversely to decrease transition rates to undesirable nodes by incorporating negative evidence. Penalized degree is in marked contrast to core normalization used in NetCore [30], which is equivalent to a biased random walk favoring transitions to high-core nodes, and by correlation, high-degree nodes, thereby exacerbating degree bias.

The new AMEND algorithm, which incorporates RWR-MH, degree bias adjustment, and B-RWR, was applied to two multi-omic datasets, each including different omic types, using multiplex-heterogeneous networks with complex configurations. The TCGA-KIRC analysis included RNA-seq, miRNA-seq, methylation, and survival data, while the OGT-KO study included transcriptomic, proteomic, phospho-proteomic, and metabolomic data. AMEND was able to recapitulate many important molecular associations with RCC, including miR-200c, miR-141, *VEGFA*, and *HIF1A*. Additionally, several enzymes involved in mitochondrial and peroxisomal lipid metabolism were captured in the module from the OGT-KO analysis, many of which corroborate previous studies of O-GlcNAc (Choline, *DECR1*, *ACSF2*).

Despite, or because of, its broad applicability, there are limitations with AMEND. Since AMEND is a tool designed to be as widely applicable as possible, with the goal of identifying densely-connected subsets of nodes with large experimental values, it may not be optimal for a specific research question or experimental design for which this is not the analytic goal. Additionally, results will be highly dependent on the choice of network. There are alternative, non-network-based frameworks for jointly analyzing multi-omic data: correlation/covariance-based (e.g., DIABLO), factor analysis-based, kernel-based, deep learning-based, and more [67]. AMEND is most suitable for complex experimental designs with noisy, disparate data, where the goal is to identify a coherent, inter-related set of biologically relevant features, and when high-quality molecular interaction data is available. Since AMEND was developed as a supervised method, it is not appropriate for the task of sample clustering.

In conclusion, we present AMEND 2.0, an active module identification method with capabilities for multi-omic data integration through multiplex-heterogeneous network diffusion, in addition to degree bias mitigation and multi-objective node selection. These capabilities make AMEND a widely applicable tool for identifying highly relevant modules that facilitate understanding of complex, heterogeneous molecular data. AMEND is easily accessible as an R package, with source code, install instructions, documentation, and examples available at https://github.com/samboyd0/AMEND.

## Supplementary Information

Additional file1: Appendix. This file acts as an appendix for the main manuscript, covering the implementation of AMEND in R, mathematical details of transition matrix construction for RWR-MH, details on degree bias adjustment methods, and further details on the AMEND-DIABLO comparison on the TCGA-KIRC data

Additional file2: Molecular Interaction Network Characteristics. Descriptions of the molecular interaction networks involved in this study, including node type, associated evaluation task, interaction type, source, species, pre-processing, and node & edge counts

Additional file3: Degree Bias Adjustment Analysis. Combinations of transition matrix types and degree bias adjustment methods compared on 2 tasks: correlation between diffusion scores and degree, and retention of high-seed-value nodes in the context of AMEND. For all figures, the restart probability is set to 0.5 for RWR. A) Correlation between diffusion scores and node degree. For each of 5 human gene expression datasets, absolute log fold changes from differential expression analysis were diffused on a human PPI network of functional and physical interactions. Pearson correlation coefficients were averaged across datasets. B) Average difference in empirical cumulative probabilities between seed values and degree for module nodes returned from AMEND. For each of 5 human gene expression datasets, seed values are absolute log fold changes from differential expression analyses assigned to nodes such that seed value and degree are perfectly negatively correlated. Results are averaged across datasets

Additional file4: ORA with GO Terms and AMEND Module from TCGA-KIRC Data. Complete list of significant Gene Ontologyterms from ORA on the AMEND module from the TCGA-KIRC data analysis. Significance level was set to 0.01 after adjustment for multiple testing using the Benjamini-Hochberg method

Additional file5: ORA with DO Terms and AMEND Module from TCGA-KIRC Data. Complete list of significant Disease Ontologyterms from ORA on the AMEND module from the TCGA-KIRC data analysis. Significance level was set to 0.01 after adjustment for multiple testing using the Benjamini-Hochberg method

Additional file6: ORA with Reactome Pathways and AMEND Module from OGT-KO Data. Complete list of significant Reactome pathways from ORA on the AMEND module from the OGT-KO data analysis. Significance level was set to 0.05 after adjustment for multiple testing using the Benjamini-Hochberg method

## Data Availability

The datasets and computer code used throughout this study are available in the following databases: Chemical-chemical links: STITCH v5 (http://stitch.embl.de/). *Mus musculus* protein-chemical links: STITCH v5 (http://stitch.embl.de/). TCGA-KIRC Methylation data: Broad GDAC Firehose (https://gdac.broadinstitute.org/). TCGA-KIRC mRNA-seq data: Broad GDAC Firehose (https://gdac.broadinstitute.org/). All other data and code: GitHub (https://github.com/samboyd0/AMEND2.0_study).
