# Supplementary material for: AMEND 2.0: module identification and multi-omic data integration with multiplex-heterogeneous graphs
Source: BMC Bioinformatics. 2025 Feb 5;26:39. doi: 10.1186/s12859-025-06063-x (PMC11800622; doi:10.1186/s12859-025-06063-x)
Supplement: Supplementary file 1 — Additional file1: Appendix. This file acts as an appendix for the main manuscript, covering the implementation of AMEND in R, mathematical details of transition matrix construction for RWR-MH, details on degree bias adjustment methods, and further details on the AMEND-DIABLO comparison on the TCGA-KIRC data [file 12859_2025_6063_MOESM1_ESM.docx]

**AMEND 2.0: Module Identification and Multi-Omic Data Integration with Multiplex-Heterogeneous Graphs**

Samuel S. Boyd*^1^, Chad Slawson^2,3,4^, Jeffrey A. Thompson^1,3^

1. Department of Biostatistics and Data Science, University of Kansas Medical Center, Kansas City, KS, 66160 USA
2. Department of Biochemistry, University of Kansas Medical Center, Kansas City, KS 66160 USA
3. University of Kansas Cancer Center, Kansas City, KS, 66160 USA
4. University of Kansas Alzheimer’s Disease Research Center, Fairway, KS, 66205 USA

**Additional File 1**

**Contents**

1. Implementing AMEND in R
2. Random Walk with Restart for Multiplex/Heterogeneous Networks
3. Degree Bias Adjustment Methods
4. DIABLO Parameter Settings for TCGA-KIRC Analysis

**Implementing AMEND in R**

The following three sub-sections will outline general steps and conventions for active module identification with the *run_AMEND()* function of the *AMEND* package in the R programming language. Complete installation instructions and package documentation can be found on the GitHub repository page (<https://github.com/samboyd0/AMEND>) or in the R programming environment with the *AMEND* package installed.

*Network Data Preparation for AMEND in R*

*AMEND* can accept three formats for network data: edge list, adjacency matrix, or *igraph* object from the *igraph* R package [1]. An edge list is a 2-column matrix where each row contains the adjacent nodes of one edge in the network, with an optional third column containing edge weights. This is perhaps the easiest to work with since many molecular interaction databases provide interaction data in edge list format by default. An adjacency matrix is an $N\times N$ matrix ($N=number of nodes$) whose $\left( i,j \right)^{th}$ element is 0 if no edge exists between node $i$ and node $j$, and a non-negative, non-zero value otherwise. An *igraph* object contains all node and edge information of a graph, along with node and edge attributes that can be easily created and manipulated.

Multiplex/heterogeneous graphs consist of multiple components and/or layers that can be input separately into *run_AMEND()*, which then creates an integrated graph from these separate graphs. These separate objects must follow a common format (edge list, adjacency matrix, or *igraph* object) and be organized as named elements of a list following certain naming conventions. Each separate graph object must be named as “component_layer” (e.g., “ppi_physical”) in order to identify that object with a specific component and layer. Input objects which correspond to bipartite graphs containing connections between different components must have component names separated by a semi-colon, *i.e.* “comp1;comp2;comp3”, or “comp1_layer1;comp2_layer1” for layer-specific bipartite connections. Due to these naming conventions, component and layer names cannot contain “_” or “;”.

Since components/layers may share common nodes with other components/layers, further naming conventions must be followed for node names in order to identify to which component and layer each node belongs. The *run_AMEND()* function will automatically append the *component_layer* names given in the network list object to the end of node names contained therein, separated by “|” . Therefore, each node in a multiplex/heterogeneous graph is identified by “name|component_layer”. Given these naming conventions, node names cannot contain “|”. For bipartite graph objects, nodes must already have component/layer info appended to them in order for the algorithm to properly identify to which part of the graph it belongs.

We will now walk through an example of network preparation for a multiplex-heterogeneous network. This hypothetical network consists of 2 components corresponding to a monoplex metabolite-metabolite interaction (MMI) component named “mmi” and a multiplex PPI component consisting of 2 layers corresponding to RNA-seq data and MS/MS proteomic data with names “ppi_rna” and “ppi_ms”. There is also a bipartite network connecting the MMI component to the PPI component. Assume each network object is in edge list format. What follows are general steps.

1. Ensure that each edge list has at least 2 columns, with the first two columns containing the nodes adjacent to each edge. If edge weights are available, ensure that all weights are between 0 and 1 and placed as the third column of the matrix.
2. For the bipartite edge list object *bp.el*, which is a matrix with column 1 corresponding to metabolites and column 2 to proteins, ensure that each name is appended with appropriate component information (e.g., ‘Choline|mmi’ or ‘TP53|ppi’). If this is not so, the following code could be used.

*bp.el[,1] <- paste(bp.el[,1], ‘mmi’, sep=’|’)*

*bp.el[,2] <- paste(bp.el[,2], ‘ppi’, sep=’|’)*

The *run_AMEND()* function will automatically connect the MMI component with both layers of the PPI component using connections in *bp.el*.

1. Create a named list object containing all of the distinct edge lists.

*input_graphs <- list(‘mmi;ppi’ = bp.el, mmi = mmi.el, ppi_rna = ppi.rna.el, ppi_ms = ppi.ms.el)*

The network data is now ready for input into *run_AMEND()*.

*Experimental Data Preparation for AMEND in R*

Experimental data are what is used by the AMEND algorithm to arrive at a context-specific active module from a static interaction network. Experimental values (e.g., log fold changes) are transformed into non-negative values to be used as seeds in RWR, which diffuse these values through the network, integrating experimental and topological information to determine node importance. For simple networks (monoplex-homogeneous), experimental data can be a named vector, with names corresponding to nodes. If the network data is an *igraph* object, this experimental information can be stored as a vertex attribute, and the name of this attribute can be supplied to the *data* argument in *run_AMEND()*.

Continuing with the multiplex-heterogeneous example, let’s assume we have log fold changes experimental data will need to be formatted as a named list corresponding to the component/layers of the network. Each element of this list should be a named vector, with names corresponding to node names and values corresponding to log fold changes. The node names in this list don’t need the *component_layer* information appended to them, as this will be done automatically by the algorithm. Not all names in the data object need to be contained in the graph, and not all nodes in the graph need to have a match in the data object. Nodes with no match will be given seed values of 0. The following code creates a list object called *input_data*, which contains all of our experimental information and will be mapped to nodes contained in the ­*input_graphs* object.comparing expression/abundance values between two groups for metabolomic, transcriptomic, and proteomic data. Since our networks are in edge list format, our

*input_data <- list(mmi = mmi.lfc, ppi_rna = rna.seq.lfc, ppi_ms = ms.lfc)*

The experimental data is now ready for input into *run_AMEND()*.

*Function Argument Specification for AMEND in R*

There are several additional arguments that need to be specified in the *run_AMEND()* function. The following will give brief descriptions of each.

- *node_type*: List object containing the names of nodes that belong to each component and layer. Unnecessary for our example, since node types will be inferred from the list names of *input_graphs*.
- *n*: Desired size of final module. The algorithm will try to approximate this size.
- *normalize*: the normalization method to use to construct the transition matrix used in RWR. One of ‘*degree*’ or *‘modified_degree’*. The latter penalizes transitions to nodes as a function of their degree.
- *k*: When *normalize=’modified_degree’*, this specifies the exponent in $D^{-k}AD^{-k}$, which modifies the adjacency matrix before degree normalization.
- *FUN*: Function(s) for transforming experimental data to be non-negative, with increasing values relating to increased importance. Several built-in functions can be referred to by character scalars (e.g., ‘exp’, ‘p_value’, ‘binary’). We will use *abs* (absolute value) from base R to indicate that we want to select for both up- and down-regulated features.
- *FUN.params*: Function parameters/arguments to be passed to functions given in *FUN*. Together, *FUN* and *FUN.params* offer much ease and flexibility in how seed values are computed.
- *brw.attr*: Values to be used for biased random walk. Should be non-negative.
- *degree.bias*: A list with two arguments: *method* to specify the degree bias adjustment method to use, and *component* to specify the components/layers it should be applied to.
- *aggregate*.*multiplex*: A list with two arguments: *primary* to specify the primary layers of multiplex components whose edges will be preferentially used during the maximum-weight connected sub-graph identification step, and *agg.method* to specify the function to aggregate diffusion scores of common nodes across layers.
- *multiplex*: Logical. For our example, this will be set to *TRUE*.
- *heterogeneous*: Logical. For our example, this will be set to *TRUE*.
- *jump.prob*: Named vector of probabilities of ‘jumping’ between different components during RWR. Lower values translate to less ‘cross-talk’ or sharing of information between components.
- *net.weight*: Named vector of weights for each component, which will be applied to the seed vectors of each component. Weights must sum to one.
- *switch.layer.prob*: Named list of vectors containing probabilities of switching between layers within a multiplex component during RWR. Lower values translate to less ‘cross-talk’ or sharing of information between layers.
- *layer.weight*: Named list of vectors of weights for each layer within a component, which will be applied to the seed vectors of each layer.

These last 4 arguments provide control over the relative importance of each network component and data type. More complete descriptions of all function arguments can be found in the *AMEND* package documentation. To bring it all together, the following will show a function call for *run_AMEND()* and what the output will contain. The result will be a named list.

*subnet <- run_AMEND(edge_list = input_graphs, n = 50, data = input_data,*

*FUN = abs, normalize = ‘degree’, multiplex = TRUE,*

*heterogeneous = TRUE, jump.prob = c(mmi = 0.25, ppi = 0.25),*

*net.weight = list(mmi = 0.5, ppi = 0.5),*

*switch.layer.prob = list(ppi = c(rna = 0.25, ms = 0.25)),*

*layer.weight = list(ppi = c(rna = 0.7, ms = 0.3)))*

*subnet$module # the final module, as an igraph object*

*subnet$score # final module score*

*subnet$stats # statistics on each iteration of the algorithm*

*subnet$time # run time*

*subnet$input_params # list of input parameters*

*subnet$subnetworks # a list of node names contained in intermediate subnetworks*

In this function call to *run_AMEND()*, we specify an approximate final module size of 50 using degree normalization. Equal weight is given to the MMI and PPI components; however, more weight is given to the RNA-seq layer of the PPI component compared to the proteomic layer, as evidenced by the *layer.weight* argument. Each component and layer has a 25 % probability for the random walker to switch/jump between different graph regions. The *subnetworks* element of the returned object will contain the nodes that were in the network at the end of each iteration. By inspecting this object, it is possible to determine where in the algorithm certain nodes were excluded or to obtain larger (or possibly smaller) subnetworks than the final module. Please consult the package documentation for more details, examples, and functions available in the *AMEND* package.

**Random Walk with Restart for Multiplex/Heterogeneous Networks**

What follows is a detailed explanation of a re-parameterization of RWR for multiplex/heterogeneous graphs that includes further generalizations compared to MultiXrank, which we call RWR-MH. The essential difference is in how the transition matrix is constructed from the adjacency matrix and in how the seed weight and cross-talk parameters are applied. The full adjacency matrix of a multiplex-heterogeneous graph is called the supra-adjacency matrix, and it comprises the sub-matrices corresponding to individual components which themselves may contain sub-matrices corresponding to individual layers (for multiplex components). The supra-adjacency matrix for a multiplex-heterogeneous graph with $N$ components is given as

$$\begin{aligned} A=\left[ \begin{matrix} A_{1} & B_{12} & \cdots& B_{1N} \\ B_{21} & A_{2} & \cdots& B_{2N} \\ \vdots& \vdots& \ddots& \vdots\\ B_{N1} & B_{N2} & \cdots& A_{N} \end{matrix} \right]\#\left( 1 \right) \end{aligned}$$

Matrices along the diagonal represent components which may include additional layers. For a multiplex component $\kappa$, its adjacency matrix is given by

$$\begin{aligned} A_{\kappa}=\left[ \begin{matrix} A_{1}^{\kappa} & C_{12}^{\kappa} & \cdots& C_{{1L}_{\kappa}}^{\kappa} \\ C_{21}^{\kappa} & A_{2}^{\kappa} & \cdots& C_{{2L}_{\kappa}}^{\kappa} \\ \vdots& \vdots& \ddots& \vdots\\ C_{L_{\kappa}1}^{\kappa} & C_{L_{\kappa}2}^{\kappa} & \cdots& A_{L_{\kappa}}^{\kappa} \end{matrix} \right]\#\left( 2 \right) \end{aligned}$$

which has $L_{\kappa}$ intra-layer adjacency matrices along the diagonal and inter-layer matrices for off-diagonal elements. The inter-layer adjacency matrix between layer $l$ and $m$ for component $\kappa$ is defined by

$$\begin{aligned} \left( C_{lm}^{\kappa} \right)_{ij}=\left\{ \begin{aligned} 1, v_{\kappa li}=v_{\kappa mj} \\ 0, o.w. \end{aligned} \right.\#\left( 3 \right) \end{aligned}$$

where $v_{kli}$ represents node $i$ from layer $l$, component $\kappa$. The bipartite adjacency matrix connecting components $\kappa$ and $\rho$ is given by

$$\begin{aligned} B_{\kappa\rho}=\left[ \begin{matrix} R_{11}^{\kappa\rho} & R_{12}^{\kappa\rho} & \cdots& R_{1L_{\rho}}^{\kappa\rho} \\ R_{21}^{\kappa\rho} & R_{22}^{\kappa\rho} & \cdots& R_{2L_{\rho}}^{\kappa\rho} \\ \vdots& \vdots& \ddots& \vdots\\ R_{L_{\kappa}1}^{\kappa\rho} & R_{L_{\kappa}2}^{\kappa\rho} & \cdots& R_{L_{\kappa}L_{\rho}}^{\kappa\rho} \end{matrix} \right]\#\left( 4 \right) \end{aligned}$$

where $R_{lm}^{\kappa\rho}$ represents the bipartite matrix between layer $l$ of component $\kappa$ and layer $m$ of component $\rho$.

The first step in constructing the transition matrix is to column-normalize sub-matrices of the supra-adjacency matrix independently. For multiplex components, this means normalizing each intra- and inter-layer matrix independently, followed by the application of between-layer cross-talk parameters such that the resulting normalized multiplex matrix is left-stochastic. An analogous process of normalization occurs at the component level, consisting of independent normalization of intra-component adjacency matrices $A_{\kappa}$ and layer-specific inter-component matrices $R_{lm}^{\kappa\rho}$. Formally, this semi-normalized supra-adjacency matrix is given by

$$\begin{aligned} M^{'}=\left[ \begin{matrix} M_{1}^{'} & S_{12}^{'} & \cdots& S_{1N}^{'} \\ S_{21}^{'} & {M^{'}}_{2} & \cdots& S_{2N}^{'} \\ \vdots& \vdots& \ddots& \vdots\\ S_{N1}^{'} & S_{N2}^{'} & \cdots& M_{N}^{'} \end{matrix} \right]\#\left( 5 \right) \end{aligned}$$

The normalized intra-component adjacency matrix $M_{\kappa}^{'}$ is defined by

$$\begin{aligned} \left( M_{\kappa}^{'} \right)_{i_{l}j_{m}}=\left\{ \begin{aligned} \frac{\left( A_{\kappa} \right)_{i_{m}j_{m}}}{\sum_{x=1}^{n_{\kappa m}} \left( A_{\kappa} \right)_{x_{m}j_{m}}}\left( 1-\delta_{\kappa m} \right) ; l=m,L_{\kappa mj}^{\kappa}\neq0,\sum_{x=1}^{n_{\kappa m}} \left( A_{\kappa} \right)_{x_{m}j_{m}}\neq0 \\ \frac{\left( A_{\kappa} \right)_{i_{m}j_{m}}}{\sum_{x=1}^{n_{\kappa m}} \left( A_{\kappa} \right)_{x_{m}j_{m}}}; l=m,L_{\kappa mj}^{\kappa}=0,\sum_{x=1}^{n_{\kappa m}} \left( A_{\kappa} \right)_{x_{m}j_{m}}\neq0 \\ \left( A_{\kappa} \right)_{i_{l}j_{m}}\cdot\frac{\delta_{\kappa m}}{L_{\kappa mj}^{\kappa}}; l\neq m,L_{\kappa mj}^{\kappa}\neq0,\sum_{x=1}^{n_{\kappa m}} \left( A_{\kappa} \right)_{x_{m}j_{m}}\neq0 \\ \left( A_{\kappa} \right)_{i_{l}j_{m}}\cdot\frac{1}{L_{\kappa mj}^{\kappa}}; l\neq m,L_{\kappa mj}^{\kappa}\neq0,\sum_{x=1}^{n_{\kappa m}} \left( A_{\kappa} \right)_{x_{m}j_{m}}=0 \\ 0 ; o.w. \end{aligned} \right.\#\left( 6 \right) \end{aligned}$$

where $\left( A_{\kappa} \right)_{i_{l}j_{m}}$ denotes the edge between the $i^{th}$ node of layer $l$ and the $j^{th}$ node of layer $m$ of component $\kappa$. The normalized bipartite adjacency matrix $S_{\kappa\rho}^{'}$ is given by

$$\begin{aligned} \left( S_{\kappa\rho}^{'} \right)_{i_{l}j_{m}}=\left\{ \begin{aligned} \frac{\left( B_{\kappa\rho} \right)_{i_{l}j_{m}}}{\sum_{x=1}^{n_{\kappa l}} \left( B_{\kappa\rho} \right)_{x_{l}j_{m}}}; \sum_{x=1}^{n_{\kappa l}} \left( B_{\kappa\rho} \right)_{x_{l}j_{m}}\neq0 \\ 0; o.w. \end{aligned} \right.\#\left( 7 \right) \end{aligned}$$

where $\left( B_{\kappa\rho} \right)_{i_{l}j_{m}}$ denotes the bipartite edge between node $i$ of layer $l$, component $\kappa$ and node $j$ of layer $m$, component $\rho$; $\delta_{\kappa m}$ is the cross-talk parameter defining the probability for the random walker of switching from layer $m$, component $\kappa$ to another layer in $\kappa$; $L_{\kappa mj}^{\kappa}$ is the number of other layers within component $\kappa$ that node $j$ of layer $m$, component $\kappa$ is connected to; lastly, $n_{\kappa l}$ is the number of nodes in layer $l$ component $\kappa$.

The final step is to apply between-component cross-talk parameters to $M^{'}$ to obtain the transition matrix $M$, formally given by

$$\begin{aligned} M=\left[ \begin{matrix} M_{1} & S_{12} & \cdots& S_{1N} \\ S_{21} & M_{2} & \cdots& S_{2N} \\ \vdots& \vdots& \ddots& \vdots\\ S_{N1} & S_{N2} & \cdots& M_{N} \end{matrix} \right]\#\left( 8 \right) \end{aligned}$$

where the transition probabilities for component $\kappa$ are defined as

$$\begin{aligned} \left( M_{\kappa} \right)_{i_{l}j_{m}}=\left\{ \begin{aligned} \left( M_{\kappa}^{'} \right)_{i_{l}j_{m}}\left( 1-\lambda_{\kappa} \right) ; \phi_{\kappa mj}\neq0 \\ \left( M_{\kappa}^{'} \right)_{i_{l}j_{m}} ; \phi_{\kappa mj}=0 \end{aligned} \right.\#\left( 9 \right) \end{aligned}$$

and bipartite transition probabilities from components $\kappa$ to $\rho$ are defined as

$$\begin{aligned} \left( S_{\rho\kappa} \right)_{ij}=\left\{ \begin{aligned} \left( S_{\rho\kappa}^{'} \right)_{i_{l}j_{m}}\cdot\frac{\lambda_{\kappa}}{\phi_{\kappa mj}\cdot L_{\kappa mj}^{\rho}} ; \phi_{\kappa mj}\neq0,L_{\kappa mj}^{\rho}\neq0,\sum_{x=1}^{n_{\kappa m}} \left( A_{\kappa} \right)_{x_{m}j_{m}}\neq0 \\ \left( S_{\rho\kappa}^{'} \right)_{i_{l}j_{m}}\cdot\frac{1}{\phi_{\kappa mj}\cdot L_{\kappa mj}^{\rho}} ; \phi_{\kappa mj}\neq0,L_{\kappa mj}^{\rho}\neq0,\sum_{x=1}^{n_{\kappa m}} \left( A_{\kappa} \right)_{x_{m}j_{m}}=0 \\ 0 ; o.w. \end{aligned} \right.\#\left( 10 \right) \end{aligned}$$

where $\lambda_{\kappa}$ is the cross-talk parameter defining the probability for the random walker of jumping from the current component $\kappa$ to another component, $\phi_{\kappa mj}$ is the number of other components that node $j$ of layer $m$, component $\kappa$ is connected to, and $L_{\kappa mj}^{\rho}$ is the number of layers in component $\rho$ that node $j$ of layer $m$, component $\kappa$ is connected to.

**Degree Bias Adjustment Methods**

This study compares three degree bias adjustment methods: Stationary Distribution Scaling (SDS), Bistochastic Scaling (BS), and Inflation-Normalization (IN). BS and IN are two novel degree bias adjustment methods that act directly on the transition matrix of a random walk, rather than by adjusting diffusion scores post-hoc. Both attempt to manipulate the transition matrix such that the entropy of its stationary distribution is maximized. The entropy of a stochastic vector $\boldsymbol{x}_{N\times1}$ is formally given by

$$\begin{aligned} E= -\sum_{i=1}^{N} x_{i}\ln\left( x_{i} \right)\#\left( 11 \right) \end{aligned}$$

and is maximized when $x_{i}=\frac{1}{N} \forall i$. Therefore, maximizing the entropy of the stationary distribution–which is a function of degree–homogenizes the influence of degree on diffusion scores. Bistochastic Scaling, as the name suggests, scales the transition matrix to be approximately bistochastic. A bistochastic matrix is a matrix with row and column sums all equal to one and possesses the property of having a principal eigenvector with maximum entropy [2]. BS relies on Iterative Proportional Fitting (IPF), a matrix scaling algorithm that scales an input matrix to have target row and column sums while preserving cross-product ratios [3]. BS employs IPF using row and column target sums all equal to one. Below is pseudocode outlining the main steps of the algorithm.

*INPUT left-stochastic matrix* $M_{N\times N}$

*SET target row and columns sums* $\boldsymbol{j}^{T}\boldsymbol{=}[1,\ldots,1]$

*SET zero-diagonal elements of* $M$ *to* $\epsilon>0$ *to aid in convergence of IPF*

*CALL IPF with initial matrix* $M$*, target row sums* $\boldsymbol{j}$*, and target column sums* $\boldsymbol{j}$*,*

*RETURNING matrix* $M^{'}$

*REDISTRIBUTE diagonal elements of* $M^{'}$ *evenly across non-zero elements of each*

*column.*

*RETURN matrix* $M_{N\times N}^{'}$

The scaled matrix $M^{'}$ can now be used in any network diffusion method relying on a transition matrix, such as RWR. By scaling the transition matrix to be approximately bistochastic, BS increases the entropy of the stationary distribution, thereby mitigating degree influence on diffusion scores.

IN relies on different mechanisms to achieve the same goal of BS, namely the maximization of the entropy of the stationary distribution. IN borrows from the inflation operator of the Markov Clustering algorithm [4]. Elements in each row of the transition matrix are raised to a certain positive power, which is a function of the stationary distribution probability associated with that row, and columns are subsequently re-normalized. This increases stationary distribution entropy by displacing incoming transition probabilities away from nodes as a function of their stationary distribution probability. Below is pseudocode outlining the main steps of the algorithm.

*INPUT left-stochastic matrix* $M_{N\times N}$

*CALCULATE stationary distribution of* $M$*,* $\boldsymbol{p}_{SD}^{T}=[s_{1},\ldots,s_{N}]$

*FOR* $k\mathbb{\in N}$*:*

*CALCULATE exponents* $\boldsymbol{e}^{T}=[e_{1},\ldots,e_{N}]$*,* $e_{j}=1+k\times s_{j}$

*SET values in each row of* $M$ *to be raised to its corresponding value in* $\boldsymbol{e}$*, such*

*that the* $i^{th}$ *row is* $[m_{i1}^{e_{i}},\ldots,m_{iN}^{e_{i}}]$

*RENORMALIZE columns of* $M$*, RETURNING* $M^{'}$

*CALCULATE stationary distribution of* $M^{'}$

*CALCULATE entropy* $E_{k}$ *of stationary distribution of* $M^{'}$

*IF* $E_{k}<E_{k-1}$*:*

*BREAK*

*END IF*

*END FOR*

*RETURN matrix* $M_{N\times N}^{'}$ *that is associated with entropy* $E_{k-1}$

In practice, a smaller grid of positive values is searched, rather than the set of natural numbers $\mathbb{N}$. As with BS, the modified matrix $M^{'}$ resulting from IN can be used in network diffusion methods such as RWR.

**DIABLO Parameter Settings for TCGA-KIRC Analysis**

This section will provide details on the parameter settings used for DIABLO [5]. We followed the recommendations of the method’s authors for choosing the number of components, which was set to 5. For the number of features from each dataset included in each factor, we set the first factor to 150, 6, and 5 for mRNA, miRNA, and methylation datasets, respectively. For the remaining four factors, we followed the recommendations of the method’s authors for parameter tuning. The design matrix was set to 0.5 for the off-diagonal elements and 0 for the diagonal elements. This represents a trade-off between selecting features with large covariance across datasets and features with good class discrimination.

**References**

1. Csárdi, G., et al., *igraph: Network Analysis and Visualization in R*. 2024.

2. Mourad, B., *On a spectral property of doubly stochastic matrices and its application to their inverse eigenvalue problem.* Linear Algebra and Its Applications, 2012. **436**(9): p. 3400-3412.

3. Fienberg, S.E., *An Iterative Procedure for Estimation in Contingency Tables.* Annals of Mathematical Statistics, 1970. **41**(3): p. 907-&.

4. Dongen, S., *Graph clustering via a discrete uncoupling process. SIAM J Matrix Anal Appl, 30:121-141.* SIAM J. Matrix Analysis Applications, 2008. **30**: p. 121-141.

5. Singh, A., et al., *DIABLO: an integrative approach for identifying key molecular drivers from multi-omics assays.* Bioinformatics, 2019. **35**(17): p. 3055-3062.
