# Supplementary figures and images for: AMEND 2.0: module identification and multi-omic data integration with multiplex-heterogeneous graphs
Source: BMC Bioinformatics. 2025 Feb 5;26:39. doi: 10.1186/s12859-025-06063-x (PMC11800622; doi:10.1186/s12859-025-06063-x)

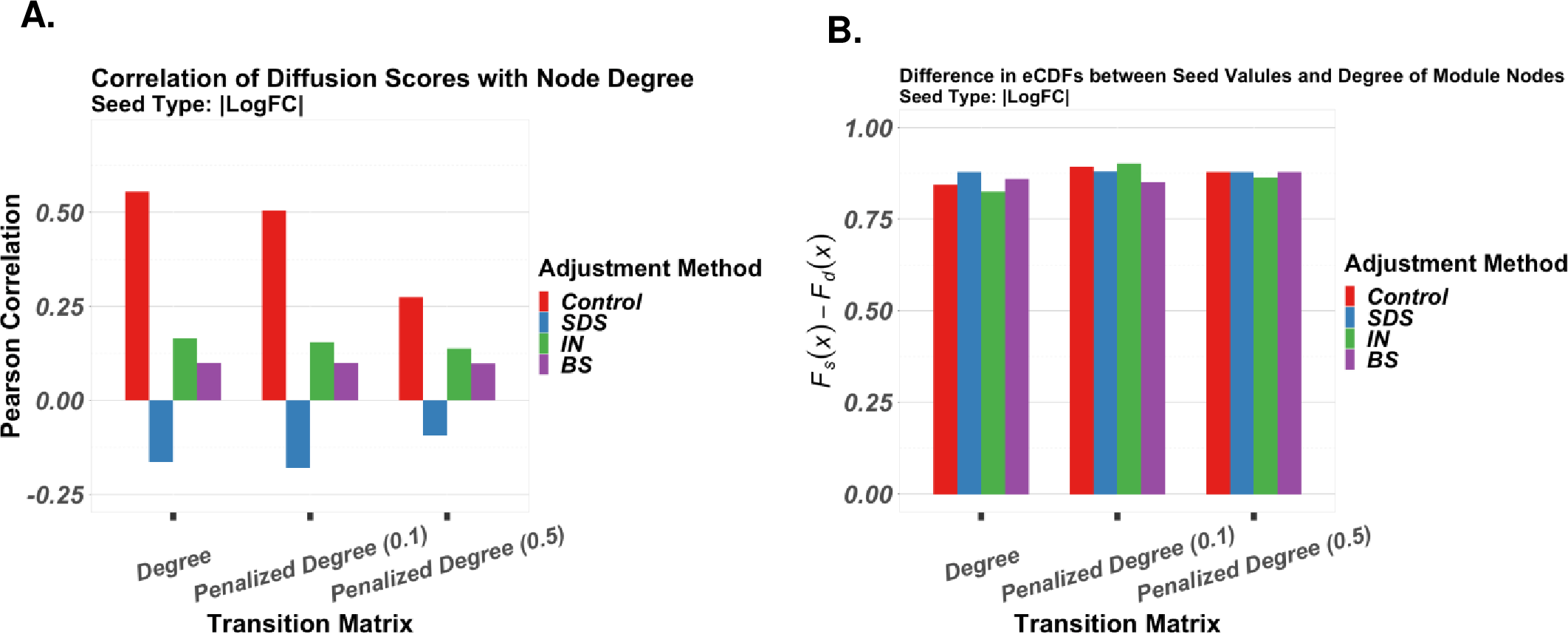

Supplement: Supplementary file 3 — Additional file3: Degree Bias Adjustment Analysis. Combinations of transition matrix types and degree bias adjustment methods compared on 2 tasks: correlation between diffusion scores and degree, and retention of high-seed-value nodes in the context of AMEND. For all figures, the restart probability is set to 0.5 for RWR. A) Correlation between diffusion scores and node degree. For each of 5 human gene expression datasets, absolute log fold changes from differential expression analysis were diffused on a human PPI network of functional and physical interactions. Pearson correlation coefficients were averaged across datasets. B) Average difference in empirical cumulative probabilities between seed values and degree for module nodes returned from AMEND. For each of 5 human gene expression datasets, seed values are absolute log fold changes from differential expression analyses assigned to nodes such that seed value and degree are perfectly negatively correlated. Results are averaged across datasets [file 12859_2025_6063_MOESM3_ESM.tif]
